# Polymer-tethered glycosylated gold nanoparticles recruit sialylated glycoproteins into their protein corona, leading to off-target lectin binding†

**DOI:** 10.1039/d2nr01818g

**Published:** 2022-08-30

**Authors:** Ashfaq Ahmad, Panagiotis G. Georgiou, Alessia Pancaro, Muhammad Hasan, Inge Nelissen, Matthew I. Gibson

**Affiliations:** Department of Chemistry, University of Warwick Gibbet Hill Road CV4 7AL Coventry UK m.i.gibson@warwick.ac.uk; Division of Biomedical Sciences, Warwick Medical School, University of Warwick Gibbet Hill Road CV4 7AL Coventry UK; Health Unit, Flemish Institute for Technological Research (VITO) Boeretang 200 Mol BE-2400 Belgium; Dynamic Bioimaging Lab, Advanced Optical Microscopy Centre and Biomedical Research Institute, Hasselt University Agoralaan C Diepenbeek BE-3590 Belgium

## Abstract

Upon exposure to biological fluids, the fouling of nanomaterial surfaces results in non-specific capture of proteins, which is particularly important when in contact with blood for *in vivo* and *ex vivo* applications. It is crucial to evaluate not just the protein components but also the glycans attached to those proteins. Polymer-tethered glycosylated gold nanoparticles have shown promise for use in biosensing/diagnostics, but the impact of the glycoprotein corona has not been established. Here we investigate how polymer-tethered glycosylated gold nanoparticles interact with serum proteins and demonstrate that the protein corona introduces new glycans and hence off-specific targeting capability. Using a panel of RAFT-derived polymers grafted to the gold surface, we show that the extent of corona formation is not dependent on the type of polymer. In lectin-binding assays, a glycan (galactose) installed on the chain-end of the polymer was available for binding even after protein corona formation. However, using sialic-acid binding lectins, it was found that there was significant off-target binding due to the large density of sialic acids introduced in the corona, confirmed by western blotting. To demonstrate the importance, we show that the nanoparticles can bind Siglec-2, an immune-relevant lectin post-corona formation. Pre-coating with (non-glycosylated) bovine serum albumin led to a significant reduction in the total glycoprotein corona. However, sufficient sialic acids were still present in the residual corona to lead to off-target binding. These results demonstrate the importance of the glycans when considering the protein corona and how ‘retention of the desired function’ does not rule out ‘installation of undesired function’ when considering the performance of glyco-nanomaterials.

## Abstract 2

Upon exposure to biological fluids, the fouling of nanomaterial surfaces results in non-specific capture of proteins, which is particularly important when in contact with blood serum for *in vivo* and *ex vivo* applications.

## Graphical abstract

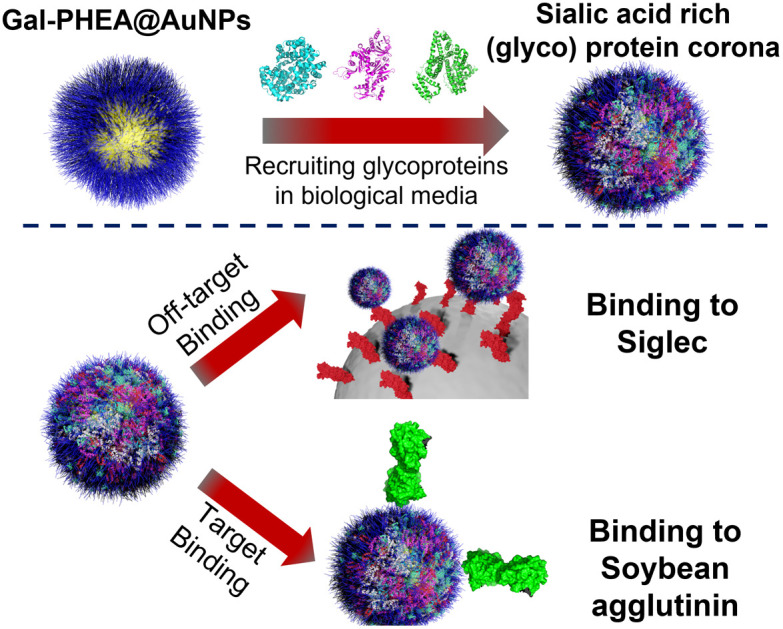

## Introduction

Cell-surface glycans within the glycocalyx are responsible for a range of biological functions from cell–cell communication and immunology to sites of pathogen adhesion.^1–5^ Aberrant glycosylation^6^ is associated with many pathologies and hyper-sialylation (for example) is exploited by tumour cells for immunosuppression.^7^ Up or downregulation of glycan-binding proteins is also associated with disease.^8^ In order to study glycan–protein interactions, as anti-adhesive therapies,^9,10^ or as biosensing/diagnostic probes, glycosylated nanomaterials have been widely explored.^11–14^ The multivalent presentation of individual glycans allows an enhancement in binding affinity from millimolar to sub nanomolar, due to the cluster glycoside effect.^15^ Glycans also bring stability to nanoparticles in complex biological media.^16^ Due to their unique plasmonic properties, gold (and silver) nanoparticles have potential in biosensing and diagnostics.^17,18^ Multivalent glyconanoparticles have also been explored as vaccine candidates^19^ or as tools for chemical glycobiology.^20,21^ Polymer-tethered sialic acid-functionalised gold nanoparticles have been designed to bind the N-terminal domain of the SARS-CoV-2 spike protein, allowing for detection in lateral flow or liquid phase assays.^22–24^ Similarly, lateral flow devices using heparin (which targets a different domain of the spike protein) have been demonstrated.^25^ In these specific examples the glycosylated nanomaterials are exposed to nasal swab elutions, which have been diluted and hence components of the biological matrix (mucins *etc.*) are also diluted. However, the situation is more complex when considering liquid biopsy (*i.e. ex vivo* blood samples) or *in vivo* applications where the glyconanoparticle is in contact with blood plasma, which contains 35–50 mg mL^−1^ of over 200 distinct proteins.^26^

Upon injection, foreign bodies such as nanoparticles are rapidly opsonised, coating the particles with a protein corona which leads to removal by macrophages.^27,28^ For drug delivery applications, PEGylation is a preferred strategy, whereby the hydrophilic nature and steric shield leads to significant enhanced circulation times, by resisting opsonisation.^27^ This corona formation can mask targeting functionality: transferrin coated nanoparticles were found to lose their targeting capacity post-corona formation, for example, which would lead to failure in a targeted drug delivery scenario.^29^ The challenge of preventing this corona is highlighted by even very dense polymer brushes, which resist certain proteins (lyzosyme/transferrin) but selectively captured albumin and immunoglobulins.^30^ The protein corona is hence of crucial consideration for any material, with cells ‘seeing’ this, rather than the underpinning nanoparticle itself, meaning the hard corona composition defines any interactions.^31^ These proteins can be described as the hard (irreversibly bound) and soft (reversibly bound) corona.^32^

Whilst the impact of the specific proteins has been widely explored, the glycome of these proteins is far less studied.^33^ It is estimated that 50% of human proteins are glycosylated, but proteomics studies do not typically capture this post-translational modification. Monopoli *et al.* showed that SiO_2_ nanoparticles, which had a hard corona, showed greater uptake by macrophages when the glycans were enzymatically removed, and increased pro-inflammatory responses.^33^ Citrate-stabilised gold nanoparticles were also shown to recruit glycosylated proteins to their surface, leading to the particles binding plant lectins.^34^ The recruitment of glycans as part of the protein corona raises essential questions about the impact on specificity/selectivity and function of glycosylated nanoparticles when in plasma. If a specific glycan is installed on the particle, is it still available, and are the observed lectin (or antibody) binding interactions due to the installed glycan or one from the non-specific glycoprotein corona? Polymer-tethered gold nanoparticles, in particular, have attracted much interest as a route to install glycans whilst introducing colloidal stability.^35–37^ The unique SPR (surface plasmon resonance) properties of gold allow colorimetric detection of lectins by aggregation, or in the case of asymmetric rods, by a shift in the local SPR peak.^12,24,38–42^ They can also be used in lateral flow diagnostics.^23,43–48^ As the polymers are typically “*grafted-to*” the particles (to enable characterisation of all individual components), this limits the grafting density for linear polymers,^49^ although emerging complex topologies (such as cycles) can lead to dense surfaces by “*grafting-to”*.^50^ This means there is likely to be exposed gold surfaces in linear grafted to glycopolymers where protein corona can form, but the impact of this has not been evaluated.

Herein, we report the recruitment of glycoproteins from plasma onto the surface of glycosylated polymer-tethered gold nanoparticles and the impact on biosensing. Using a library of galactosylated poly(*N*-hydroxyethylacrylamide) tethers it is shown that the glycoprotein corona introduces significant amounts of sialic acids. This corona did not remove the underpinning binding function of the particles, which could lead to the assumption that it had no, or minor, impact. However, a panel of sialic acid-binding lectins, including Siglecs, was found to bind the particles post-corona formation. Blocking the surface before addition to plasma reduced the magnitude of the glycoprotein corona, but the off-target binding capacity was retained. These results show the importance of tuning the glyco-interface if plasmonic nanoparticles are to be deployed in blood-contacting applications and appropriate choice of controls when testing specificity.

## Results and discussion

**Fig. 1:**
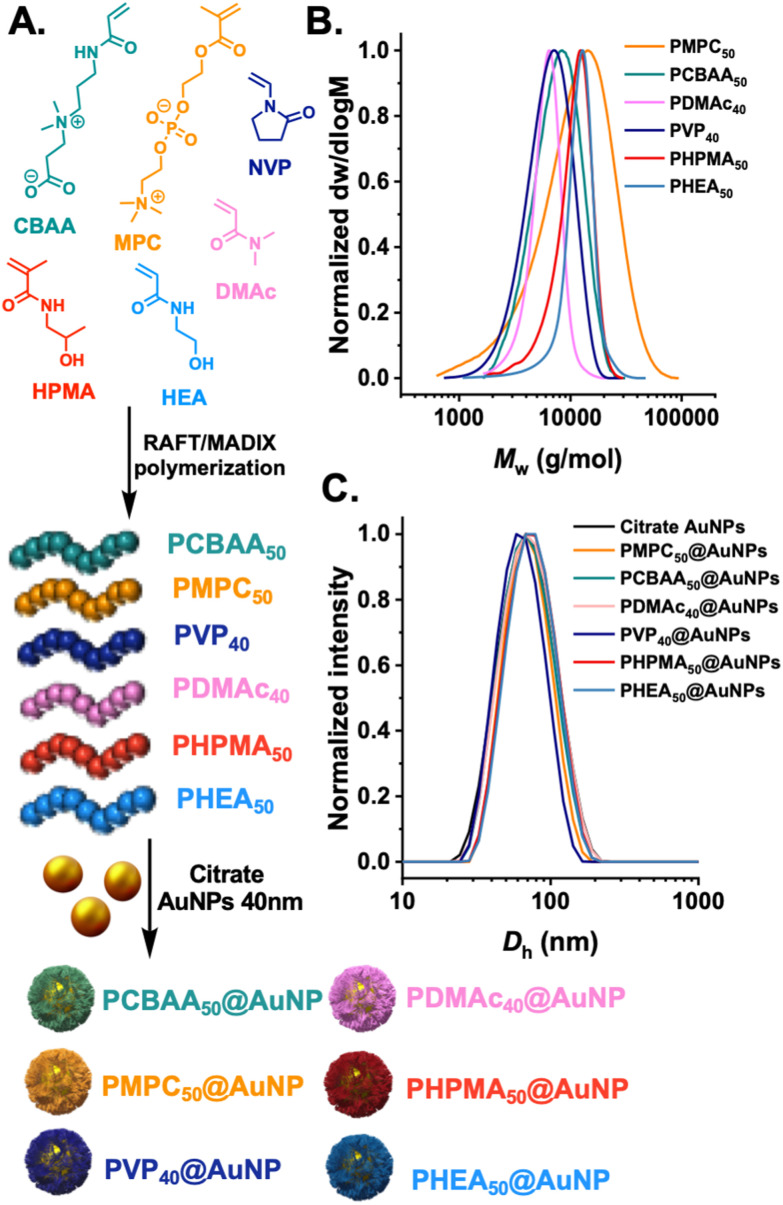
Synthesis of polymer-tethered gold nanoparticles. (A) Polymers synthesised in this study. Subscript denotes the targeted degree of polymerisation; (B) size exclusion chromatography of the polymer library; (C) dynamic light scattering of citrate AuNPs (40 nm) and with polymers tethered to their surface.

The first step was to synthesize a panel of polymers, to evaluate protein corona formation on gold nanoparticles synthesised by the “*grafting-to*” approach. We anticipated that as this approach gives relatively low surface grafting densities, the nature of the polymer tether would be relatively less important than in high-grafting density nanoparticles, so long as the polymers themselves have net neutral charge. The “*grafting-to”* approach was selected as we have previously demonstrated to be a viable and useful method to obtain glycosylated gold nanoparticles, which can be deployed in (non-blood contacting) sensing applications.^22,23,38^ RAFT (reversible addition–fragmentation chain-transfer) polymerisation was employed as this installed a masked thiol at the *α*-terminal for subsequent immobilisation onto a pre-formed gold nanoparticles, Fig. 1A. Each monomer type required a different appropriate RAFT (or MADIX) agent to provide control and the details of this are in the ESI.† The polymers were all characterised by ^1^H-NMR analysis (Fig. S1–S6†) and size exclusion chromatography (SEC), revealing mono-model distributions, Fig. 1B and Table 1. The indicated DP (in subscript) is the targeted DP from the [monomer] : [CTA] ratio. The polymers were subsequently immobilised onto 40 nm citrate-coated gold nanoparticles using an established procedure,^35^ with centrifugation/resuspension cycles used to remove excess polymer. The particles were then analysed by dynamic light scattering (DLS) and zeta potential, showing single peak distributions for all the polymer coatings and negative surface charge as expected for this class of nanomaterials, Fig. 1C.

**Table 1:** Polymers synthesised by RAFT (or MADIX) polymerisation and polymer-coated gold nanoparticle characterisation

Sample	*M*_n, SEC RI_ a (g mol^−1^)	*Đ*_M_ a	*D*_h_ b (nm)	PD^b^	Zeta-potential^c^ (mV)
PMPC_50_	7700	1.85	—	—	—
PCBAA_50_	6600	1.25	—	—	—
PDMAc_40_	4800	1.10	—	—	—
PVP_40_	4600	1.30	—	—	—
PHPMA_50_	9400	1.19	—	—	—
PHEA_50_	9300	1.14	—	—	—
Bare gold 40 nm	—	—	61.3 ± 3.7	0.18 ± 0.01	−37.7 ± 3.1
PMPC_50_@AuNP_40_	—	—	88.1 ± 4.1	0.43 ± 0.05	−25.4 ± 1.0
PCBAA_50_@AuNP_40_	—	—	65.1 ± 1.5	0.20 ± 0.01	19.5 ± 0.9
PDMAc_40_@AuNP_40_	—	—	64.4 ± 0.8	0.17 ± 0.01	−25.4 ± 2.3
PVP_40_@AuNP_40_	—	—	58.3 ± 1.1	0.11 ± 0.01	−28.4 ± 1.6
PHPMA_50_@AuNP_40_	—	—	67.4 ± 1.8	0.13 ± 0.01	−13.5 ± 5.1
PHEA_50_@AuNP_40_	—	—	68.7 ± 1.6	0.12 ± 0.01	−17.5 ± 2.0

a *M*_n_ and *Đ*_M_ values calculated from PMMA standards using 5 mM NH_4_BF_4_ in DMF as the eluent.

b *D*_h_ and PD values determined by DLS (the error represents the standard deviation from 5 repeat measurements).

c Zeta-potential values measured from microelectrophoretic analysis at pH = 7.

**Fig. 2:**
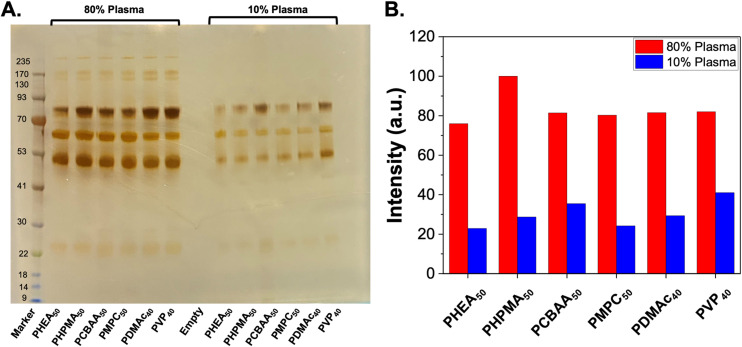
SDS-PAGE of protein corona formation on polymer-coated gold nanoparticles. (A) Silver-stained gel showing hard corona proteins released from nanoparticles after incubation with bovine plasma. (B) Densitometry analysis of gel. Polymer codes refer to polymer coatings (Fig. 1) on 40 nm gold particles.

To first validate protein corona formation, naked (citrate-capped) AuNPs were incubated with bovine plasma at various dilutions (10%, 50% and 80%). The AuNPs were then isolated by centrifugation and subjected to washing. The soft corona (proteins which are released into the supernatant after each wash) as well as the hard corona (those which remain on the particle after washing) were measured.^32^ Protein binding was evaluated by sodium dodecyl sulfate–polyacrylamide gel electrophoresis (SDS-PAGE) under reducing conditions (Fig. S9†). In these initial experiments it was observed that Coomassie staining did not give sufficient resolution due to low amount of the soft and hard corona proteins, which could give the impression of no protein corona. Hence silver staining was used to increase the detection limits of this assay which enabled the visualisation of the soft (reversibly bound) and hard (irreversibly bound) corona components,^51^ and is shown in the ESI (Fig. S9 and S10†). Using this method, the panel of polymer-coated AuNPs was exposed to 10% and 80% bovine plasma, and the total hard corona was observed using silver-staining. Densitometry analysis of the gel showed that all the polymers used here lead to similar hard corona formation at both plasma densities and similar pattern of proteins (judged verses a protein ladder control), Fig. 2. This initial screen confirmed that in the “*grafting-to*” scenario, all the polymers used gave essentially the same hard-corona formation, suggesting that the surface coverage, rather than polymer identity was the key factor here. However, it should be noted that different densities of each polymer on the surface would be achieved, which do impact their glycan-binding outputs^38^ (the overall aim of this study). It should also be noted that we selected uncharged polymers (and a betaine, with no net-charge), to reduce non-specific protein interactions, and different results would be expected with charged polymer coating. Further quantitative analysis of the corona is included later in this manuscript.

**Fig. 3:**
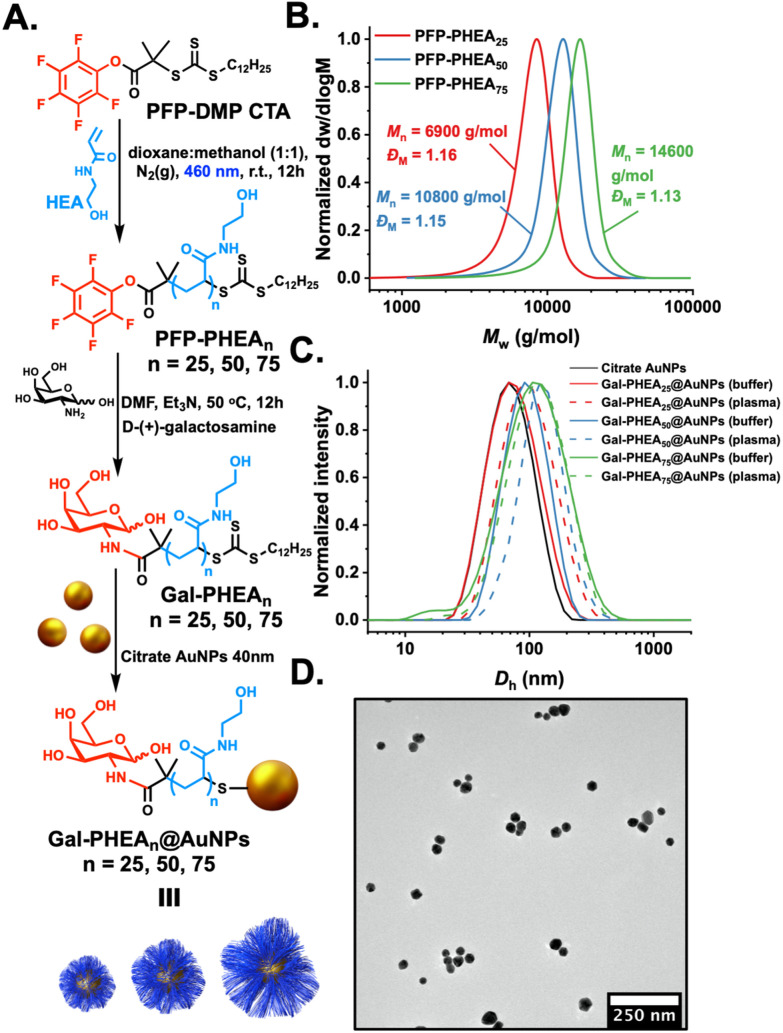
Glyconanoparticle synthesis. (A) Synthetic scheme for the synthesis of PFP-terminated PHEA, functionalisation with galactosamine and immobilisation onto gold nanoparticles; (B) size exclusion chromatography analysis of PHEA's; (C) dynamic light scattering of polymer-coated particles in buffer and in plasma; (D) representative TEM of polymer-coated nanoparticles. AuNPs = 40 nm in all cases.

With confirmation of the protein corona formation, its impact on the underpinning lectin-binding capacity of glycosylated nanoparticles could be evaluated. Poly(hydroxyethyl acrylamide), PHEA, was taken forward for this study as all the polymers showed similar protein corona formation patterns, and PHEA has been demonstrated to be a good ligand for glycan installation for biosensing^22,43^ and serum incubation has been shown to impact the binding outcomes in a nanorod based assay.^38^ 2-Deoxy, 2-amino-galactose was installed onto PHEA polymers using an established procedure,^35^ displacing a *ω*-terminal pentafluorophenyl group, as shown in Fig. 3A, confirmed by ^19^F nuclear magnetic resonance (NMR) and Fourier transform infrared (FTIR) analysis (Fig. S7 and S8†). The PHEAs were also characterised by SEC, Fig. 3B, revealing monomodal distributions. Resultant galactose-terminated polymer ligands were immobilised onto 40 nm AuNPs, as described above, to give a library of glycosylated, polymer-coated nanoparticles, confirmed by DLS. Polymer and nanoparticle characteristics are reported in Table 2. Fig. 3D shows a representative TEM (transmission electron microscopy) image of the polymer-coated particles. Fig. 3C shows that upon incubation in both buffer (PBS) and plasma solutions that the particles were stable against aggregation which is essential for their lectin biding studies (below) where aggregation is used as the (positive) signal detection output. The silver-stained gel and densitometry analysis showing the formation of hard corona on the surface of Gal-PHEA_*n*_@AuNPs (*n* = 25, 50, 75) is provided in ESI,† with buffer incubated particles as a negative control (Fig. S11†).

**Table 2:** Polymers synthesised by RAFT (or MADIX) polymerisation and polymer-coated gold nanoparticle characterisation

Sample	*M*_n, SEC RI_ a (g mol^−1^)	*Đ*_M_ a	*D*_h_ b (nm)	PD^b^	Zeta-potential^c^ (mV)
PFP-PHEA_25_	6900	1.15	—	—	—
PFP-PHEA_50_	10 800	1.14	—	—	—
PFP-PHEA_75_	14 600	1.13	—	—	—
Gal-PHEA_25_@AuNP_40_ (buffer)	—	—	68.5 ± 0.6	0.21 ± 0.01	−22.3 ± 1.9
Gal-PHEA_25_@AuNP_40_ (plasma)	—	—	88.1 ± 0.9	0.19 ± 0.01	−17.8 ± 0.8
Gal-PHEA_50_@AuNP_40_ (buffer)	—	—	71.2 ± 0.4	0.21 ± 0.01	−23.6 ± 4.2
Gal-PHEA_50_@AuNP_40_ (plasma)	—	—	70.8 ± 0.5	0.16 ± 0.01	−19.3 ± 0.5
Gal-PHEA_75_@AuNP_40_ (buffer)	—	—	89.3 ± 1.3	0.24 ± 0.03	−20.5 ± 0.3
Gal-PHEA_75_@AuNP_40_ (plasma)	—	—	119.7 ± 5.6	0.21 ± 0.13	−14.9 ± 0.9

a *M*_n_ and *Đ*_M_ values calculated from PMMA standards using 5 mM NH_4_BF_4_ in DMF as the eluent.

b *D*_h_ and PD values determined by DLS (the error represents the standard deviation from 5 repeat measurements).

c Zeta-potential values measured from microelectrophoretic analysis at pH = 7.

**Fig. 4:**
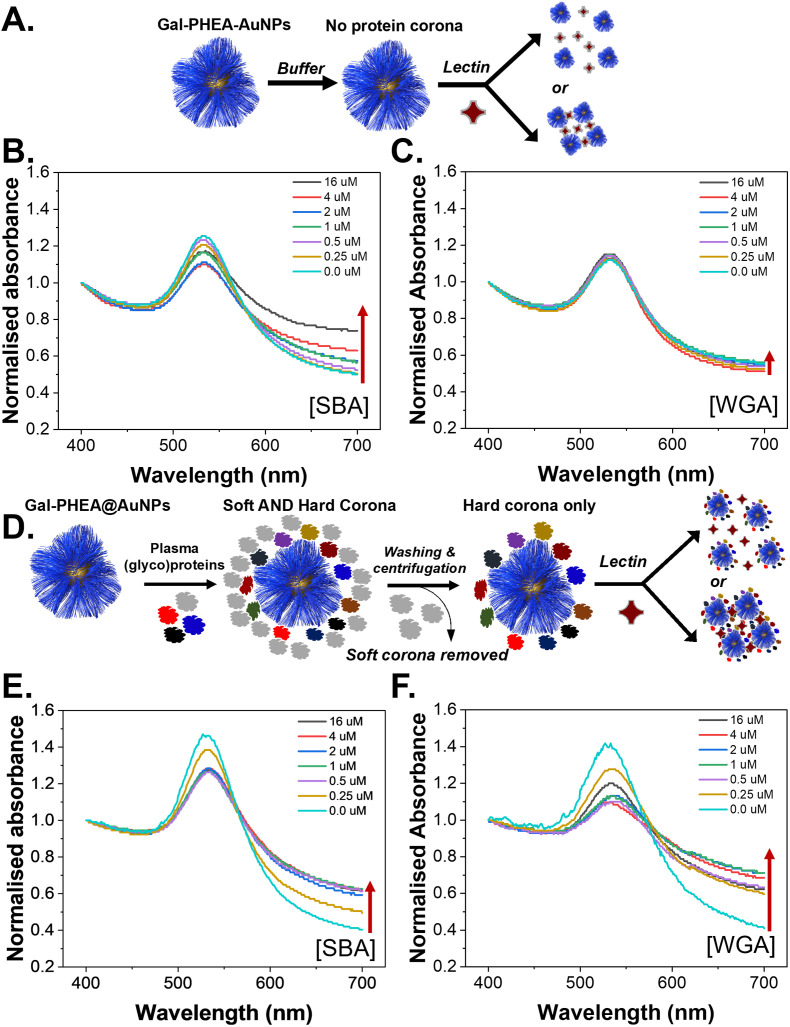
Effect of hard corona on lectin binding capacity to Gal-PHEA_25_@AuNPs. (A) Schematic of aggregation assay in buffer only; (B) UV-Vis spectra verses SBA in buffer; (C) UV-vis spectra verses WGA in buffer; (D) schematic of hard corona formation and aggregation assay; (E) UV-Vis spectra verses SBA with hard corona; (F) UV-Vis spectra verses WGA with hard corona.

To evaluate the role of the (glyco)protein corona on lectin binding by the glycosylated particles a colorimetric aggregation assay was used (Fig. 4A). Lectin cross-linking (as many lectins have multiple binding sites) of glycosylated nanoparticles leads to a red-blue colour shift due to coupling of their SPR bands, Fig. 4B, which can be detected using UV-Visible spectroscopy. As expected, addition of SBA (soybean agglutinin) lead to aggregation of the glycosylated AuNPs in a dose-dependent manner, and the polymer chain length controlled the extent of this.^35^ A negative control of WGA (wheat germ agglutinin) was also used and there was no, or very small, changes in the UV-Vis spectra, Fig. 4C, consistent with no binding, as would be expected for WGA which has no affinity towards galactosamine, but can bind sialic acids (see below).^52,53^ With these controls in hand, plasma-incubated nanoparticles were then subject to the same lectins and analysed, Fig. 4D. In the case of SBA (galactosamine binding) there was a reduction in the extent of binding, Fig. 4E. Taken alone this would suggest that the polymer-tethered nanoparticles can retain some function after formation despite the protein corona. However, upon addition of WGA (which did not interact with the buffer-only nanoparticles and does not bind galactose) a new interaction was seen, with aggregation occurring, Fig. 4F. WGA has affinity towards terminal sialic acid units^53^ and hence this suggests that the protein corona has introduced an additional off-specific binding interaction which could compromise performance. Similar results were seen for all chain lengths of particles, and their UV-Vis traces are included in the ESI (Fig. S12–S14†). It is important to note that the chain length and gold core size affect the magnitudes of binding responses due to differences in aggregation extent.^35^

**Fig. 5:**
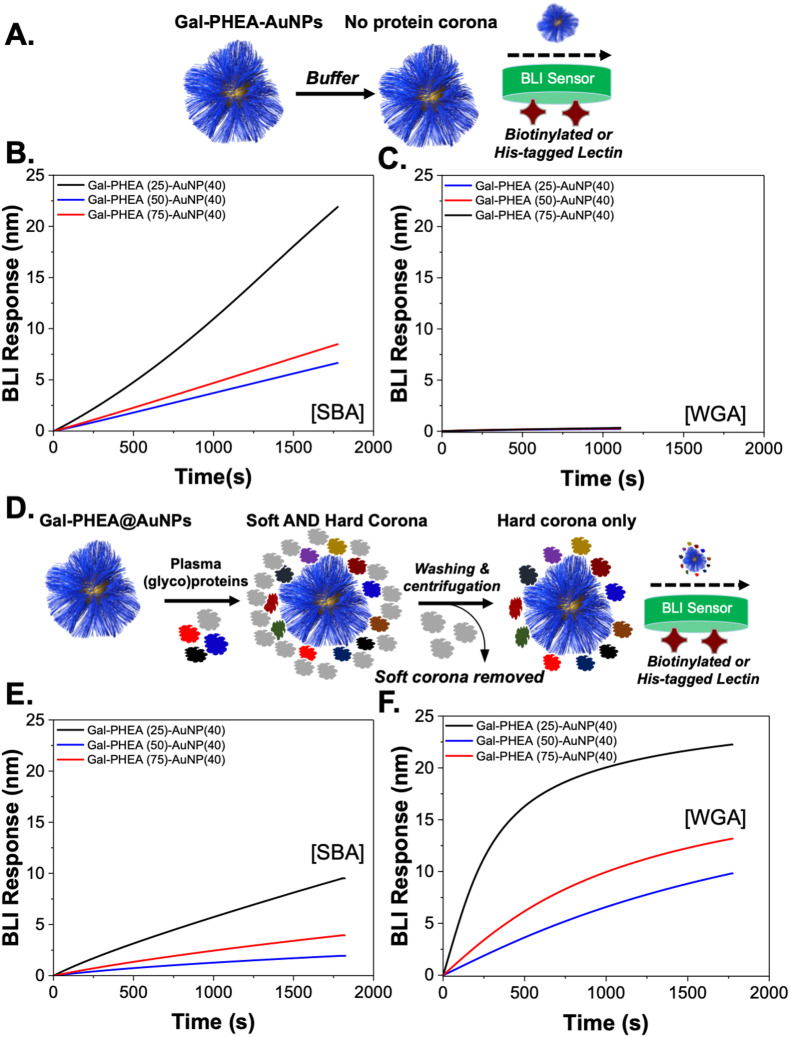
Impact of corona on lectin binding using biolayer interferometry (BLI). (A) Schematic of buffer-only lectin binding; (B) glyco-nanoparticles binding to SBA in buffer only; (C) glyco-nanoparticles binding to WGA in buffer only; (D) schematic of hard-corona coated particles lectin binding; (E) glyco-nanoparticles binding to SBA with hard corona; (F) glyco-nanoparticles binding to WGA with hard corona.

To further validate the new binding interactions from the protein corona, a second assay format was used, biolayer interferometry (BLI), which can detect binding of multivalent glyco-nanoparticles even when aggregation (*e.g.* due to sterics or low density) does not occur.^36,54^ Biotinylated SBA and WGA were immobilised onto streptavidin (SA) coated BLI sensors and the glyco-nanoparticles with different polymer chain lengths were exposed, Fig. 5A. An increase in signal indicates particle binding. It should be noted that due to the size of the particles, and multivalency, dissociation is rarely seen, so only the association phase is used here as a screen for binding, with the different lectin specificities providing an internal control. It was also not possible to obtain complete curves for SBA due to the high affinity, but the association to the lectin was clear. Fig. 5B and C show the particles in buffer, against SBA and WGA, respectively. As seen in the aggregation assay there was clear binding towards SBA but no interaction against WGA. This confirms the aggregation assays; the particles do not have intrinsic affinity towards WGA. However, upon pre-exposure to bovine plasma proteins very different behaviour is seen, Fig. 5D. The plasma exposure reduced the extent of SBA binding by approximately 50% but introduced significant binding towards WGA, Fig. 5E and F. This is a significant observation as if only the target lectin, and a non-lectin, negative control is used, one could summarize that lectin binding is not impacted by the protein corona, but this analysis shows the opposite and could have major implications for biosensing in liquid biopsies or in drug-delivery/imaging applications.

**Fig. 6:**
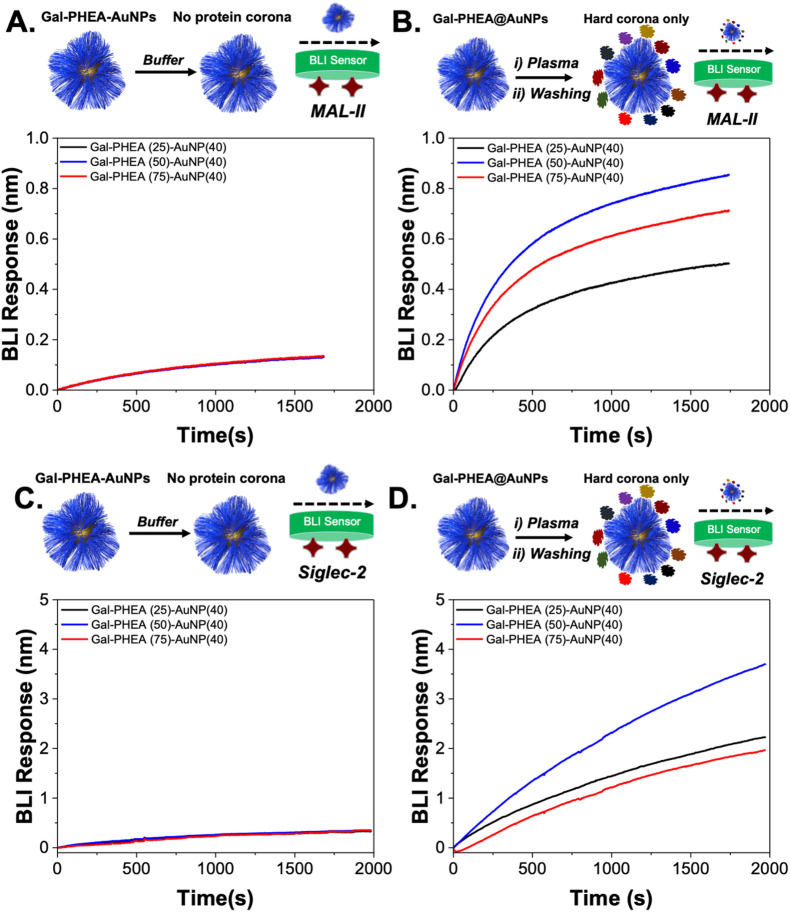
Impact of corona on sialic acid binding lectins, using BLI. (A) Glyco-nanoparticles verses MAL-II in buffer alone; (B) glyco-nanoparticles with hard corona verses MAL-II; (C) glyco-nanoparticles verses Siglec-2 in buffer alone; (D) glyco-nanoparticles with hard corona verses Siglec-2.

WGA is known to bind (*N*-acetylglucosamine) GlcNAc and also sialic acids, so an additional lectin was explored to confirm the above findings. MAL II (*Maackia amurensis* lectin) was chosen which has affinity towards terminal 2,3-sialic acids.^55^Fig. 6 shows the results of this, showing that in the buffer only system there was no binding. When the particles were incubated with bovine plasma, significant binding was observed providing further evidence that sialic acids are being introduced to the glyco-nanoparticles surface due to the protein corona. To demonstrate the biomedical relevance of these observations, the interaction with Siglec-2 (sialic acid-binding immunoglobulin-type lectin 2, also known as CD22) which is found on B cells (in the immune system) and has affinity for 2,6 linked sialosides was undertaken.^56,57^ The silver-stained gel and densitometry analysis showing the formation of hard corona on the surface of glyconanoparticles after incubation with plasma is provided in ESI (Fig. S15†). Before plasma incubation the glyco-nanoparticles showed no significant binding to immobilised Siglec-2, Fig. 6C. Post-plasma incubation there was significant binding to the Siglec-2, Fig. 6D, which in a biomedical context would be an undesirable side effect if a glyco-nanoparticles was used for *e.g.* imaging. If liquid biopsy of plasma were being used for diagnostics, this would lead to potential false positives. It should be noted, that other applications such as glyco-nanoparticles for us in nasal swabs, may not be impacted so heavily as the background matrix will be distinct.^23,24^

**Fig. 7:**
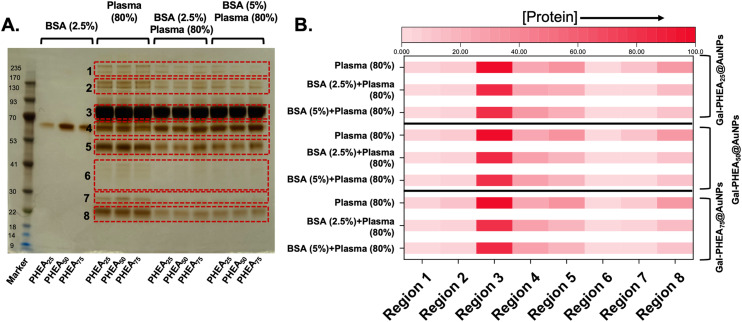
SDS-PAGE of protein corona formation on Gal-PHEA_*n*_-coated (*n* = 25, 50, 75) nanoparticles with and without BSA blocking. (A) Silver-stained gel showing hard corona proteins released from nanoparticles; (B) densitometry analysis of gel shown as a heat map. Polymer codes refer to polymer coatings (Fig. 2) on 40 nm gold particles.

A commonly used blocking method in molecular biology, designed to ‘coat’ exposed high energy surfaces which would otherwise capture protein, is to add bovine serum albumin, BSA.^58^ [Note, de-glycosylated BSA was used here]. Therefore, to evaluate if BSA blocking is sufficient to prevent or reduce the capture of sialylated proteins, gel electrophoresis was used. Particles were first incubated with BSA, and then isolated, before being placed into serum, as described above. Silver staining of the gel showed that pre-incubation with BSA did decrease the overall protein level (as BSA by definition during block is now present on the surface), Fig. 7.

**Fig. 8:**
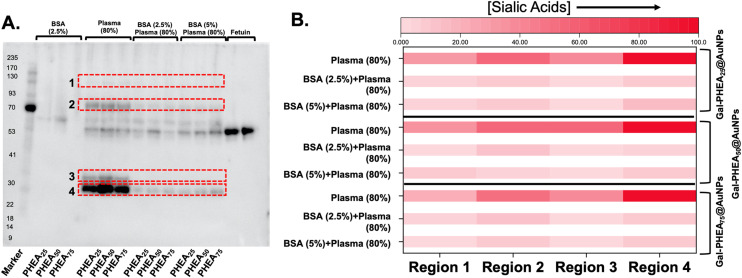
Western blot analysis of sialic acid contents of the glycoproteins corona after exposure of nanoparticles to bovine plasma. (A) Western blot using SiaFind Pan-Specific Lectenz, with darker regions indicating more sialic acids; (B) densitometry analysis presented as heat map showing change in total sialic acid recruitment to the particles with/out blocking.

BSA blocking was not expected to reduce the total amount of protein fouling (as the BSA itself is on the particle), but to prevent the introduction of sialylated proteins from plasma. Therefore, sialic acid-binding recombinant engineered proteins (SiaFind Pan-Specific Lectenz which bind sialoglycans terminated by Sia-α2,3-Gal, Sia-α2,6-Gal, and Sia-α2,8-Sia)^59^ were employed to qualify the changes in sialyation. Fig. 8 shows the results of western blotting, with fetuin (a sialylated protein) as a positive control and deglycosylated BSA as the negative. Incubation of the particle with bovine plasma alone showed significant staining indicative of sialic acids. Pre-incubation of the particles, however, with BSA lead to a significant reduction in signal. In particular, the sialic acids in region 3 were completely removed for all particles, and others decreased by 5 to 10-fold, as determined by densitometry (Fig. S16 and S17†).

**Fig. 9:**
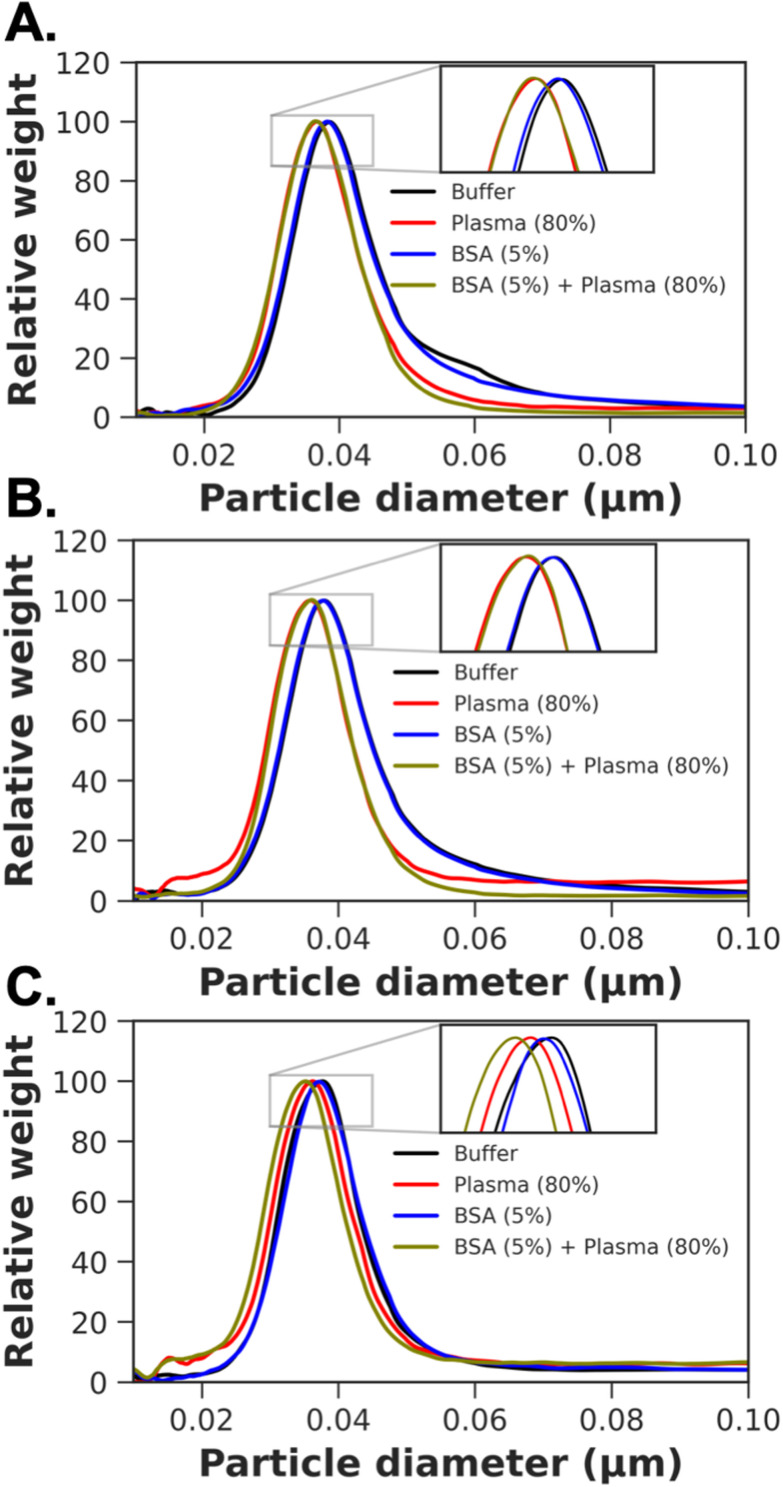
Differential centrifugation sedimentation analysis of particles, in buffer, plasma, BSA, or BSA then plasma. (A) Gal-PHEA_25_@AuNP_40_; (B) Gal-PHEA_50_@AuNP_40_; (C) Gal-PHEA_75_@AuNP_40_.

To provide further insight into the protein corona formation, differential centrifugal sedimentation (DCS) was employed. The DCS is a simple technique that can be utilised as a high-precision tool for the reliable characterisation of nanoparticles’ size distribution even in biological environment. For particles of a known density, the particles’ diameter is calculated using modified Stokes’ law based on the sedimentation time of a particle through a sucrose gradient present in a spinning disc. Here, we considered high-density metallic core AuNPs with a lower-density shell of biomolecules. The shell thickness can be calculated from the shift in particle mobility between particles before and after corona formation, if the size and density of the core nanoparticle are known and the density of the corona can be estimated,^60,61^ and reported in Table 3. In the case of PHEA 25 and 50, pre-incubation with BSA lead to a small reduction in the total amount of plasma captured but an increase was seen for the degree of polymerisation (DP) of 75, Fig. 9. This confirms the gel electrophoresis measurements that BSA blocking does impact the distribution of the proteins which are captured but does not prevent all fouling.

**Table 3:** Differential centrifugal sedimentation analysis of protein binding to particles. All measurements were performed in triplicate (*N* = 3, mean ± SD). A core–shell mathematical model was used to calculate the coating thickness (nm) reported in this table^61^

	BSA only	Plasma only	BSA then plasma
Gal-PHEA_25_@AuNPs	0.24 ± 0.08	2.33 ± 0.10	2.36 ± 0.11
Gal-PHEA_50_@AuNPs	0.26 ± 0.18	2.29 ± 0.24	2.17 ± 0.20
Gal-PHEA_75_@AuNPs	0.36 ± 0.09	2.02 ± 0.19	2.55 ± 0.11

**Fig. 10:**
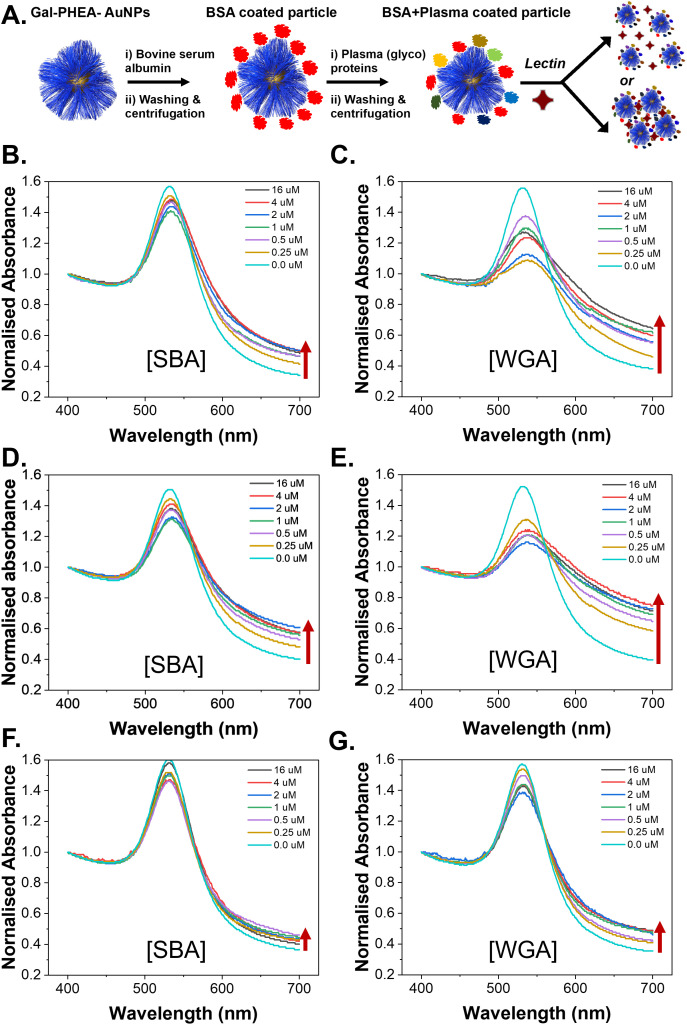
Lectin binding by aggregation assay against nanoparticles pre-blocked with BSA, before exposure to plasma. (A) Schematic of experimental process; (B) Gal-PHEA_25_@AuNP_40_ verses SBA; (C) Gal-PHEA_25_@AuNP_40_ verses WGA; (D) Gal-PHEA_50_@AuNP_40_ verses SBA; (E) Gal-PHEA_50_@AuNP_40_ verses WGA; (F) Gal-PHEA_75_@AuNP_40_ verses SBA; (G) Gal-PHEA_75_@AuNP_40_ verses WGA.

With the information that BSA blocking can reduce the amount of sialic acid-associated glycoproteins on the particles the aggregation assays were again repeated using blocked nanoparticles, Fig. 10. In all cases the SBA binding was retained post BSA/plasma treatment with similar trends in terms of the impact of polymer chain length as seen for pristine particles. The retention of binding was greater than seen using plasma incubation only. However, despite the decrease in sialic acid content, there was significant interaction with the WGA, showing that sufficient sialic acids are still being introduced to lead to off-target binding. This is a significant observation as it shows that even with the very large reduction in binding by BSA blocking, the small number of retained sialic acids are enough to induce some off-specific effects.

The data presented above reveals that glycosylated nanoparticles prepared by the “*grafting to”* approach recruit significant amounts of glycans (as glycoproteins) to their surface when exposed to plasma. These additional glycans lead to off-target binding which if the intention was drug delivery, for example, would compromise performance and highlights the need for glyco-analysis, as well as protein analysis, or any nanomaterial which will contact plasma (or other biological fluids, which are not explored here). One solution to this could be an enzymatic deglycosylation step (which would not remove glycans with unnatural linkages) as shown by Monopoli *et al.*^33^ Particles prepared by “*grafting-from”* would produce higher densities and hence less surface vacancies for fouling, but have the downside that quality control and analytics of *e.g.* polymer length is more challenging, which is a crucial parameter in glyco-nanoparticles performance.^49^

## Conclusions

Here we report the crucial importance of evaluating the presence of a protein corona on polymer-coated gold nanoparticles and the functional impact of the introduction of glycoproteins to the particle, which leads to significant off-target effects not detected by proteomic analysis alone. A range of uncharged synthetic polymers was prepared and immobilised onto gold nanoparticles, inspired by the importance of gold nanoparticles in existing (*e.g.* lateral flow) and emerging (plasmonic) bioassays or imaging/drug delivery. It was found that the total amount of protein corona was not dependent on the polymer type, attributed to the relatively low grafting density in a “*grafting-to”* scenario, meaning the vacant surface sites can recruit protein. Galactosylated particles were then studied for protein corona formation and crucially lectin binding. The particles retained their binding towards a lectin partner (soybean agglutinin) before and after exposure to plasma proteins. This could be interpreted that the protein corona is not impacting their performance. However, by interrogating the particles with a secondary lectin that also binds sialic acids (wheat germ agglutinin), it was observed that the glyco-nanoparticles could engage other sialic acid-binding lectins, post-plasma incubation, confirmed using MAL-II also. The findings were confirmed by aggregation and biolayer interferometry analysis. The biomedical importance was demonstrated by showing that particles incubated in human serum led to off-target binding to human Siglec-2. The use of (deglycosylated) bovine serum albumin as a blocking agent was evaluated. It was found to reduce specific fractions of the protein corona using gel electrophoresis, but differential centrifugation sedimentation analysis showed only minor changes in the total amount of protein captured. The results show firstly that any measure of the impact of protein corona formation in a nanomaterial must include glycan-binding analysis of some type, particularly sialic acids, given their prevalence in the human glycoproteome. Secondly, we show that a conventional blocking agent does not prevent the introduction of sufficient sialic acid to introduce off-specific binding events. If this class of particles were to be used in blood-contacting applications, alternative blocking strategies are required. However, this does not prevent their use in other application areas where plasma proteins are not present or at lower concentrations and will be the subject of further study

## Experimental section

### Materials

All chemicals were used as supplied unless otherwise stated. d-(+)-Galactosamine hydrochloride (99%), 2-(dodecylthiocarbonothioylthio)-2-methylpropionic acid (98%, DMP), 2-(dodecylthiocarbonothioylthio)-2-methylpropionic acid pentafluorophenyl ester (98%, PFP-DMP), triethylamine (TEA, ≥99%), methyl 2-bromopropionate (98%), carbon disulfide (anhydrous, ≥99%), 2-bromopropionic acid (≥99%), 2,2′-azobis(2-methylpropionitrile) (98%, AIBN) and monomers *N*-(2-hydroxypropyl)methacrylamide (99%, HPMA), *N*-hydroxyethyl acrylamide (97%, HEA), 2-methacryloyloxyethyl phosphorylcholine (97%, MPC) and *N*-vinyl-2-pyrrolidinone (≥99%, NVP) were all purchased from Sigma-Aldrich. The monomer *N*,*N*-dimethylacrylamide (≥99%, DMAC), was also purchased from Sigma Aldrich and passed through a column of basic alumina to remove inhibitor prior to use. Monomer 3-[(3-acrylamidopropyl)dimethylammonio]propanoate (>95%, CBAA) was purchased from TCI chemicals. Citrate stabilised gold nanoparticles (AuNPs, OD = 1) of 40 nm diameter, human serum (Heat Inactivated, Sterile Filtered, Product H3667), bovine plasma (P4639), globulins free bovine serum albumin (A3059), HEPES buffer and phosphate buffered saline (PBS) tablets were also purchased from Sigma-Aldrich. Sodium chloride (≥99.5%) and calcium chloride were purchased from Thermo Fisher Scientific. Chain transfer agents (CTAs) of 2-(ethoxycarbonothioyl)sulfanyl propanoate (EXEP) and 2-(((butylthio)carbonothiolyl)thio)propanoic acid were synthesised according to previously described processes.^62–64^ Octet® Streptavidin (18-5019) and NTA Biosensors (18-5101) were purchased from Sartorius. 96-Well black flat bottom microplates were obtained from Greiner Bio-One Ltd (655209). The unconjugated soybean agglutinin (SBA), wheat germ agglutinin (WGA), and *Maackia amurensis* lectin II (MAL II) lectins were obtained from Vector Laboratories. Human Siglec-2/CD22 lectin was purchased from ACRO Biosystems (CD2-H52H8). The EZ-Link™ Sulfo-NHS-LC-Biotin reagent for biotinylating lectins was purchased from Thermo Fisher Scientific. SiaFind™ Pan-Specific Lectenz® Kit (SK0501) was purchased from Lectenz Bio. Formvar-carbon coated copper grids were purchased from EM Resolutions. Clear and black half area 96-well plates were purchased from Greiner Bio-one. Photo-polymerisation reactions were conducted using an EvoluChem™ PhotoRedOx Temperature Controlled Box fitted with an EvoluChem™ LED spotlight (P201-18-2 450–455 nm) with total irradiance of 30 mW cm^−2^ and light beam angle of 25° operating at a wavelength of *λ* = 450–455 nm. All experiments were conducted using Milli-Q grade water (resistivity of 18.2 mΩ cm at 25 °C, 4 ppb total organic carbon).

### Example polymerisation: photo-polymerisation of *N*-vinylpyrrolidone using 2-(ethoxycarbonothioyl)sulfanyl propanoate

2-(Ethoxycarbonothioyl)sulfanyl propanoate (EXEP) (0.15 g, 0.72 mmol, 1 eq.) and *N*-vinylpyrrolidone (NVP) (8 g (7.7 mL), 72 mmol, 100 eq.) were dissolved in 2.04 mL of dioxane in a vial. Resulting solution was degassed by sparging with N_2_(g) for 15 min and the sealed vial was incubated at 37 °C with magnetic stirring under 460 nm light irradiation for 4 h. After that time, polymerisation was quenched by removing sealing and exposing it to air. An aliquot of crude polymerisation mixture was withdrawn for ^1^H NMR in methanol-*d*_4_ for conversion and *M*_n, NMR_ analysis. The reaction was rapidly cooled in liquid nitrogen and precipitated into diethyl ether. The polymer was re-precipitated into diethyl ether from dioxane twice to yield a pale-yellow polymer product that was further dried under vacuum. *M*_n, NMR_ was calculated by end-group analysis by comparing the integrations of the –CH_3_ signals (t, 1.42 ppm) of methyl end-group with those of the corresponding signals of the –CH signal (d, 3.69–4.02 ppm) of polymer backbone. ^1^H NMR (400 MHz, methanol-*d*_4_): *δ* (ppm) = 4.02–3.69 (br d, C***H*** of polymer backbone), 3.49–3.13 (br m, NC***H***_**2**_CH_2_ of polymer side chain), 2.54–2.17 (br m, NC(O)C***H***_**2**_ of polymer side chain), 2.17–1.94 (br s, NCH_2_C***H***_**2**_ of polymer side chain), 1.93–1.45 (br d, CHC***H***_**2**_ of polymer backbone), 1.42 (t, 3H, C***H***_**3**_CH_2_O). *M*_n, NMR_ = 4700 g mol^−1^ (DP_PVP, NMR_ = 41). SEC (5 mM NH_4_BF_4_ in DMF) *M*_n, SEC RI_ = 4600 g mol^−1^, *Đ*_M, SEC RI_ = 1.30.

### End-group modification of PFP-poly(*N*-hydroxyethyl acrylamide) (PFP-PHEA) homopolymers using galactosamine

In a typical reaction, PFP-PHEA_25_ (100 mg, 0.011 mmol), galactosamine (11.4 mg, 0.053 mmol) were dissolved in 5 mL DMF with 0.05 M triethylamine (TEA) (50 μL). The reaction was stirred at 50 °C for 16 h. The polymer was precipitated into diethyl ether from methanol three times and dried over under vacuum. ^19^F-NMR and IR analysis were performed and confirmed the loss of the pentafluoro end-group. Same procedure was followed for the synthesis of Gal-PHEA_50_ and Gal-PHEA_75_.

### Gold nanoparticle functionalization

Approximately 1 mg of the desired polymer was added to a micro-centrifuge tube and dissolved in 100 μL of high-purity water. 900 μL of the citrate-stabilised gold nanoparticle solution was added to this tube (40 nm NP solution) that was then agitated for 30 min in the absence of light. To remove excess polymer, the particles were centrifuged and following careful removal of the supernatant, the particles were then redispersed in 1 mL of MilliQ water, and the centrifugation-resuspension process repeated for a total of 3 cycles. After the final cycle the particles were dispersed in 1 mL of MilliQ water for future use. TEM, DLS and zeta-potential analyses were performed on the samples after dilution to an appropriate analysis concentration.

### Formation of biomolecular corona

Plasma was reconstituted in ultrapure water and filtered through a 0.22 μm syringe filter. The filtered plasma was aliquoted into 2 ml cryo-tubes and stored −80 °C until use. For formation of biomolecular corona, an aliquot of the plasma from −80 °C was first defrosted at room temperature and then diluted to a protein concentration of 10% or 80% (v/v) with pure water. This concentration was close to the *in vitro and in vivo* plasma protein concentration in cell culture and blood, respectively. For the assay, 250 μL of the gold nanoparticles (∼1.0 final OD) were incubated with an equal volume (1 : 1) of the diluted plasma for 1 hour at 37 °C, and 300 rpm. The unbound plasma proteins were removed by centrifugation at 12 000*g*, 20 °C for 20 min. The supernatant, containing unbound plasma proteins, were discarded and pellet, containing gold nanoparticles-plasma complex, was resuspended in 400 μL PBS and centrifuged again. The washing step was repeated three times to ensure removal of soft corona proteins *i.e.*, loosely bound proteins. After each centrifugation step, 10 μL of the supernatant (containing the released soft corona proteins) was collected for SDS-PAGE analysis. After the final wash, the hard-corona coated gold nanoparticles were either resuspended in NuPAGE™ LDS Sample Buffer (2×) (containing 50 mM DTT) for SDS-PAGE analysis or resuspended in desired volume of assay buffer (10 mM HEPES, 150 mM NaCl, 10 mM, 10 mM CaCl_2_) for lectin binding assays.

## Data availability

Any additional research data supporting this publication can be found in the Supporting Information or at https://wrap.warwick.ac.uk.

## Conflicts of interest

The authors declare no conflict of interest.

## Supplementary Material

NR-014-D2NR01818G-s001
