# Supplementary material for: Polymer-tethered glycosylated gold nanoparticles recruit sialylated glycoproteins into their protein corona, leading to off-target lectin binding
Source: Nanoscale. 2022 Aug 30;14(36):13261–73. doi: 10.1039/d2nr01818g (PMC9494357; doi:10.1039/d2nr01818g)
Supplement: NR-014-D2NR01818G-s001 [file NR-014-D2NR01818G-s001.pdf]

Electronic Supplementary Material (ESI) for Nanoscale.
This journal is © The Royal Society of Chemistry 2022

Supporting Information

"Polymer Tethered Glycosylated Gold Nanoparticles Recruit Sialylated Glycoproteins into their Protein Corona, Leading to Off-Target Lectin Binding"

*Ashfaq Ahmad,[a] Panagiotis G. Georgiou,[a] Alessia Pancaro,[c,d] Muhammad Hasan,[a] Inge Nelissen[c,d] and Matthew I. Gibson[a,b]**

[a] Department of Chemistry, University of Warwick, Gibbet Hill Road, CV4 7AL, Coventry, UK

[b] Warwick Medical School, University of Warwick, Gibbet Hill Road, CV4 7AL, Coventry, UK

[c] Health Unit, Flemish Institute for Technological Research (VITO), Boeretang 200, Mol, BE-2400, Belgium.

[d] Dynamic Bioimaging Lab, Advanced Optical Microscopy Centre and Biomedical Research Institute, Hasselt University, Agoralaan C, Diepenbeek, BE-3590, Belgium

*Corresponding Author: m.i.gibson@warwick.ac.uk (M.I.G.)

Material and methods

Materials

All chemicals were used as supplied unless otherwise stated. D-(+)-galactosamine hydrochloride (99%), 2-(dodecylthiocarbonothioylthio)-2-methylpropionic acid (98%, DMP), 2-(dodecylthiocarbonothioylthio)-2-methylpropionic acid pentafluorophenyl ester (98%, PFP-DMP), triethylamine (TEA, ≥99%), methyl 2-bromopropionate (98%), carbon disulfide (anhydrous, ≥99%), 2-bromopropionic acid (≥99%), 2,2′-azobis(2-methylpropionitrile) (98%, AIBN) and monomers *N*-(2-hydroxypropyl)methacrylamide (99%, HPMA), *N*-hydroxyethyl acrylamide (97%, HEA), 2-methacryloyloxyethyl phosphorylcholine (97%, MPC) and *N*-vinyl-2-pyrrolidinone (≥99%, NVP) were all purchased from Sigma-Aldrich. The monomer *N*,*N*-dimethylacrylamide (≥99%, DMAC), was also purchased from Sigma Aldrich and passed through a column of basic alumina to remove inhibitor prior to use. Monomer 3-[(3-acrylamidopropyl)dimethylammonio]propanoate (>95%, CBAA) was purchased from TCI chemicals. Citrate stabilised gold nanoparticles (AuNPs, OD = 1) of 40 nm diameter, human serum (H3667), bovine plasma (P4639), globulins free bovine serum albumin (A3059), HEPES buffer and phosphate buffered saline (PBS) tablets were also purchased from Sigma-Aldrich. Sodium chloride (≥99.5%) and calcium chloride were purchased from Thermo Fisher Scientific. Chain transfer agents (CTAs) of 2-(ethoxycarbonothioyl)sulfanyl propanoate (EXEP) and 2-(((butylthio)carbonothiolyl)thio)propanoic acid were synthesised according to previously described processes.[1–3] Octet® Streptavidin (18-5019) and NTA Biosensors (18-5101) were purchased from Sartorius. 96-Well black flat bottom microplates were obtained from Greiner Bio-One Ltd (655209). The unconjugated soybean agglutinin (SBA), wheat germ agglutinin (WGA), and *Maackia amurensis* lectin II (MAL II) lectins were obtained from Vector Laboratories. Human Siglec-2/CD22 lectin was purchased from ACRO Biosystems (CD2-H52H8). The EZ-Link™ Sulfo-NHS-LC-Biotin reagent for biotinylating lectins was purchased from Thermo Fisher Scientific. SiaFind™ Pan-Specific Lectenz® Kit (SK0501) was purchased from Lectenz Bio. Formvar-carbon coated copper grids were purchased from EM Resolutions. Clear and black half area 96-well plates were purchased from Greiner Bio-one. Photo-polymerisation reactions were conducted using an EvoluChem™ PhotoRedOx Temperature Controlled Box fitted with an EvoluChem™ LED spotlight (P201-18-2 450-455 nm) with total irradiance of 30 mW.cm^{-2} and light beam angle of 25° operating at a wavelength of λ = 450–455 nm. All experiments were conducted using Milli-Q grade water (resistivity of 18.2 mΩ cm at 25 °C, 4 ppb total organic carbon).

Methods

Analytical and Physical Methods

NMR Spectroscopy. ^{1}H-NMR and ^{19}F-NMR spectra were recorded at 300 MHz or 400 MHz on a Bruker DPX-300 or DPX-400 spectrometer respectively, with methanol-d_4 as the solvent. Chemical shifts of protons are reported as δ in parts per million (ppm) and are relative to solvent residual peak (CH_3OH, δ = 3.31 ppm/ DMSO, δ = 2.50 ppm).

FT-IR Spectroscopy. Fourier Transform-Infrared (FT-IR) spectroscopy measurements were carried out using an Agilent Cary 630 FT-IR spectrometer, in the range of 650 to 4000 cm^{-1}.

Size Exclusion Chromatography in DMF. Size exclusion chromatography (SEC) analysis was performed on an Agilent Infinity II MDS instrument equipped with differential refractive index (DRI), viscometry (VS), dual angle light scatter (LS) and variable wavelength UV detectors. The system was equipped with 2 x PLgel Mixed D columns (300 x 7.5 mm) and a PLgel 5 µm guard column. The mobile phase used was DMF (HPLC grade) containing 5 mM NH_4BF_4 at 50 ^{o}C at flow rate of 1.0 $mL.min^{-1}$. Poly(methyl methacrylate) (PMMA) standards (Agilent EasyVials) were used for calibration between 955,000 – 550 $g.mol^{-1}$. Analyte samples were filtered through a nylon membrane with 0.22 μm pore size before injection. Number average molecular weights (M_n), weight average molecular weights (M_w) and dispersities ($Đ_M = M_w/M_n$) were determined by conventional calibration and universal calibration using Agilent GPC/SEC software.

Aqueous Size Exclusion Chromatography. SEC analysis of poly(2-methacryloyloxyethyl phosphorylcholine) (PMPC) and poly(carboxybetaine acrylamide) (PCBAA) homopolymers was performed on an Agilent Technologies Infinity 1260 MDS instrument equipped with a differential refractive index (DRI), light scattering (LS) and viscometry (VS) detectors. The column set used were Tosoh TSKGel GPWXL *2. The mobile phase used was 0.1 M $NaNO_3$. Column oven and detector temperatures were regulated to 40°C, flow rate 1 mL/min. Poly(ethyleneoxide) standards (Agilent EasyVials) were used for calibration between 1,368,000 – 106 $g.mol^{-1}$. Analyte samples were filtered through a hydrophilic GVWP membrane with 0.22 µm pore size before injection. Number average molecular weights (M_n), weight average molecular weights (M_w) and dispersities ($Đ_M = M_w/M_n$) were determined by conventional calibration and universal calibration using Agilent GPC/SEC software.

Dynamic Light Scattering (DLS). Hydrodynamic diameters (D_h) and size distributions of particles were determined by dynamic light scattering using a Malvern Zetasizer Nano ZS with a 4 mW He-Ne 633 nm laser module operating at 25 °C. Measurements were carried out at an angle of 173° (back scattering), and results were analysed using Malvern DTS 7.03 software. All determinations were repeated 5 times with at least 10 measurements recorded for each run. D_h values were calculated using the Stokes-Einstein equation where particles are assumed to be spherical.

Zeta Potential Analysis. Zeta potential was measured by the technique of microelectrophoresis, using a Malvern Zetasizer Nano ZS instrument, at room temperature at 633 nm. All reported measurements were the average of at least five runs. Zeta potential was calculated from the corresponding electrophoretic mobilities (μ_E) by using the Henry's correction of the Smoluchowski equation ($\mu_E = 4\pi\ \varepsilon_0\ \varepsilon_r\ \zeta\ (1+\kappa r)/6\pi\ \mu$).

UV-Vis Spectroscopy. Absorbance measurements BioTek Epoch microplate reader in the wavelength (λ) range of 400-700 nm (step = 5 nm).

Differential centrifugal sedimentation (DCS). Differential centrifugal sedimentation (DCS) was performed using a CPS DC24000 disc centrifuge with 8–24% (w/w) sucrose gradient and a rotation speed of 24000 RPM. A fresh sucrose gradient was prepared after each condition (plasma, BSA, BSA + plasma). Before each run, polyvinyl chloride latex beads (483 nm) with narrow size distribution were used as calibration standard to ensure accuracy of the measurements. All measurements were performed in triplicate. The binding of biomolecules onto the gold nanoparticles' surface increases the particles' size but lowers their overall density. The CPS analysis assumes a constant particle density, so over-estimating the particle density means an under-estimate of the particle size.[4,5] For this reason, the binding of polymers or biomolecules to the gold nanoparticles results in an apparent decrease in the particle size reported by CPS. A core–shell mathematical model was used to analyse the coating thickness of the gold nanoparticles as previously described after the glycopolymers functionalisation and for each sample (Gal-$PHEA_{25}$@$AuNP_{40}$; Gal-$PHEA_{50}$@$AuNP_{40}$; Gal-$PHEA_{75}$@$AuNP_{40}$) in the different conditions.[6–8]

Transmission Electron Microscopy. Dry-state TEM imaging was performed on a JEOL JEM-2100Plus microscope operating at an acceleration voltage of 200 kV. All dry-state samples were diluted with deionised water and then deposited onto formvar-coated copper grids.

Experimental Procedures

Synthetic Procedures

Photo-polymerisation of *N*-vinylpyrrolidone using 2-(ethoxycarbonothioyl)sulfanyl propanoate.

2-(ethoxycarbonothioyl)sulfanyl propanoate (EXEP) (0.15 g, 0.72 mmol, 1 eq) and *N*-vinylpyrrolidone (NVP) (8 g (7.7 mL), 72 mmol, 100 eq) were dissolved in 2.04 mL of dioxane in a vial. Resulting solution was degassed by sparging with N_2(g) for 15 min and the sealed vial was incubated at 37°C with magnetic stirring under 460 nm light irradiation for 4h. After that time, polymerisation was quenched by removing sealing and exposing it to air. An aliquot of crude polymerisation mixture was withdrawn for ^{1}H NMR in methanol-d_4 for conversion and $M_{n,NMR}$ analysis. The reaction was rapidly cooled in liquid nitrogen and precipitated into diethyl ether. The polymer was re-precipitated into diethyl ether from dioxane twice to yield a pale-yellow polymer product that was further dried under vacuum. $M_{n,NMR}$ was calculated by end-group analysis by comparing the integrations of the –CH_3 signals (t, 1.42 ppm) of methyl end-group with those of the corresponding signals of the –CH signal (d, 3.69-4.02 ppm) of polymer backbone. ^{1}H NMR (400 MHz, methanol-d_4): δ (ppm) = 4.02-3.69 (br d, C***H*** of polymer backbone), 3.49-3.13 (br m, NC$\boldsymbol{H_2}$$CH_2$ of polymer side chain), 2.54-2.17 (br m, NC(O)C$\boldsymbol{H_2}$ of polymer side chain), 2.17-1.94 (br s, NCH_2C$\boldsymbol{H_2}$ of polymer side chain), 1.93-1.45 (br d, CHC$\boldsymbol{H_2}$ of polymer backbone), 1.42 (t, 3H, C$\boldsymbol{H_3}$$CH_2$O). $M_{n,NMR}$ = 4700 g mol^{-1} ($DP_{PVP, NMR}$ = 41). SEC (5 mM NH_4BF_4 in DMF) $M_{n, SEC RI}$= 4600 g mol^{-1}, $Đ_{M, SEC RI}$ = 1.30.

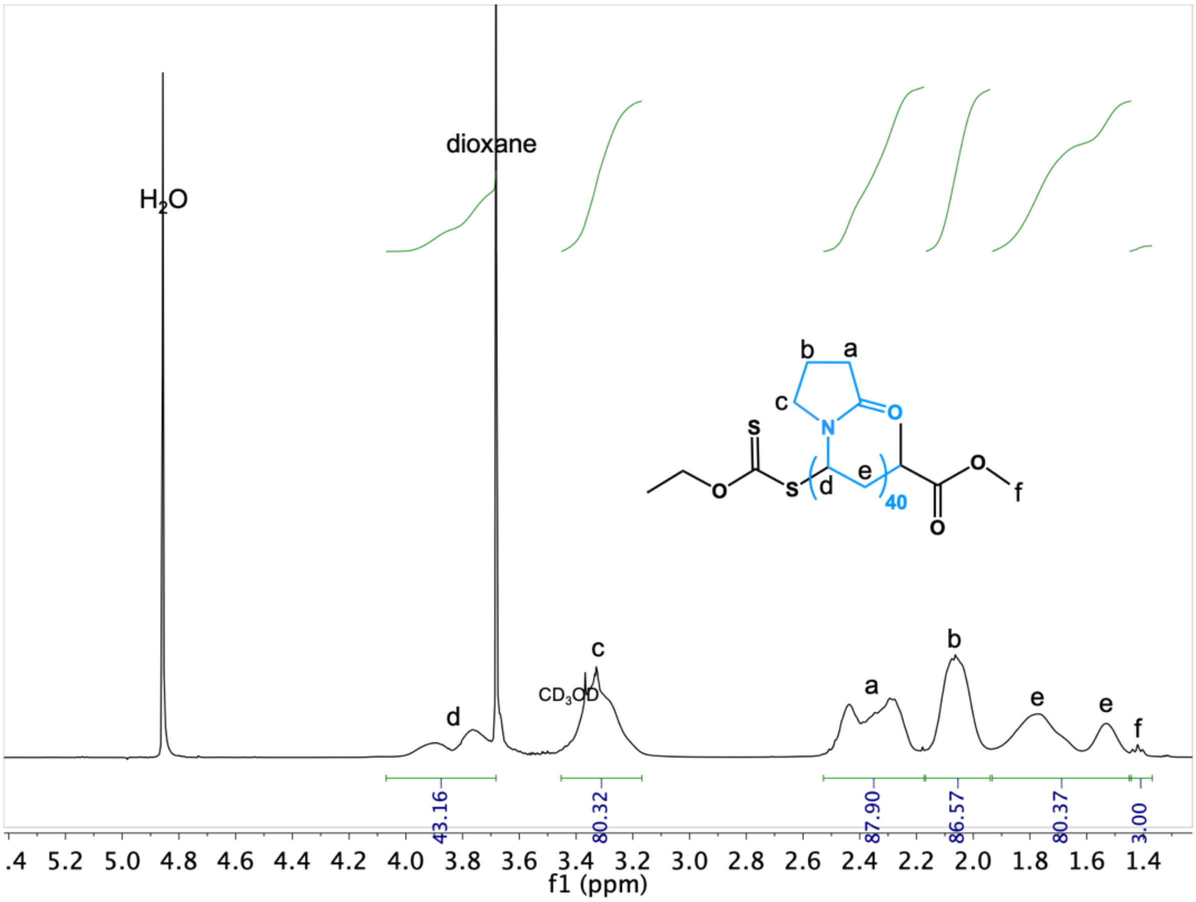

Figure S1. ^{1}H NMR spectrum for poly(vinyl pyrrolidinone), PVP_{40} homopolymer recorded in methanol-d_4.

Photo-polymerisation of 2-methacryloyloxyethyl phosphorylcholine (MPC) using 2-(dodecylthiocarbonothioylthio)-2-methylpropionic acid.

A vial was charged with 2-(dodecylthiocarbonothioylthio)-2-methylpropionic acid (50 mg, 0.14 mmol, 1 eq), MPC (2.02 g, 6.86 mmol, 50 eq), and 10 mL of methanol. The vial was then sealed and deoxygenated using three successive cycles of freeze-pump-thaw to remove O_2(g). The sealed vial was incubated at 37°C with magnetic stirring under 460 nm light irradiation for 12h. The polymerisation was quenched by exposing the vial to air. An aliquot was withdrawn for determination of monomer conversion by ^{1}H NMR spectroscopy. The polymer was precipitated into acetone from methanol to yield a sticky yellow polymer product and dialysed against dionised water for 72h (MWCO = 1 kDa) and subsequently freeze-dried to yield a pale-yellow solid. $M_{n,NMR}$ by end-group analysis could not be calculated due to overlapping signals of the –CH_3 of methyl end-group with those of the corresponding polymer signals. ^{1}H NMR (400 MHz, methanol-d_4): δ (ppm) = 4.51-4.05 (br t, $OC\boldsymbol{H_2}C\boldsymbol{H_2}OPOC\boldsymbol{H_2}$ of polymer side chain), 3.90-3.75 (br s, $C\boldsymbol{H_2}N(CH_3)_3$ of polymer side chain), 3.45-3.15 (br s, $CH_2N(C\boldsymbol{H_3})_3$ of

polymer side chain), 2.20-1.65 (br m, $CH_2C(CH_3)$ of polymer backbone), 1.40-0.85 (br m, $CH_2C(C\boldsymbol{H_3})$ of polymer backbone). SEC (0.1 M $NaNO_3$ in H_2O) $M_{n,\ \mathrm{SEC\ RI}}$ = 7700 g mol^{-1}, $Đ_{\mathrm{M,\ SEC\ RI}}$ = 1.85.

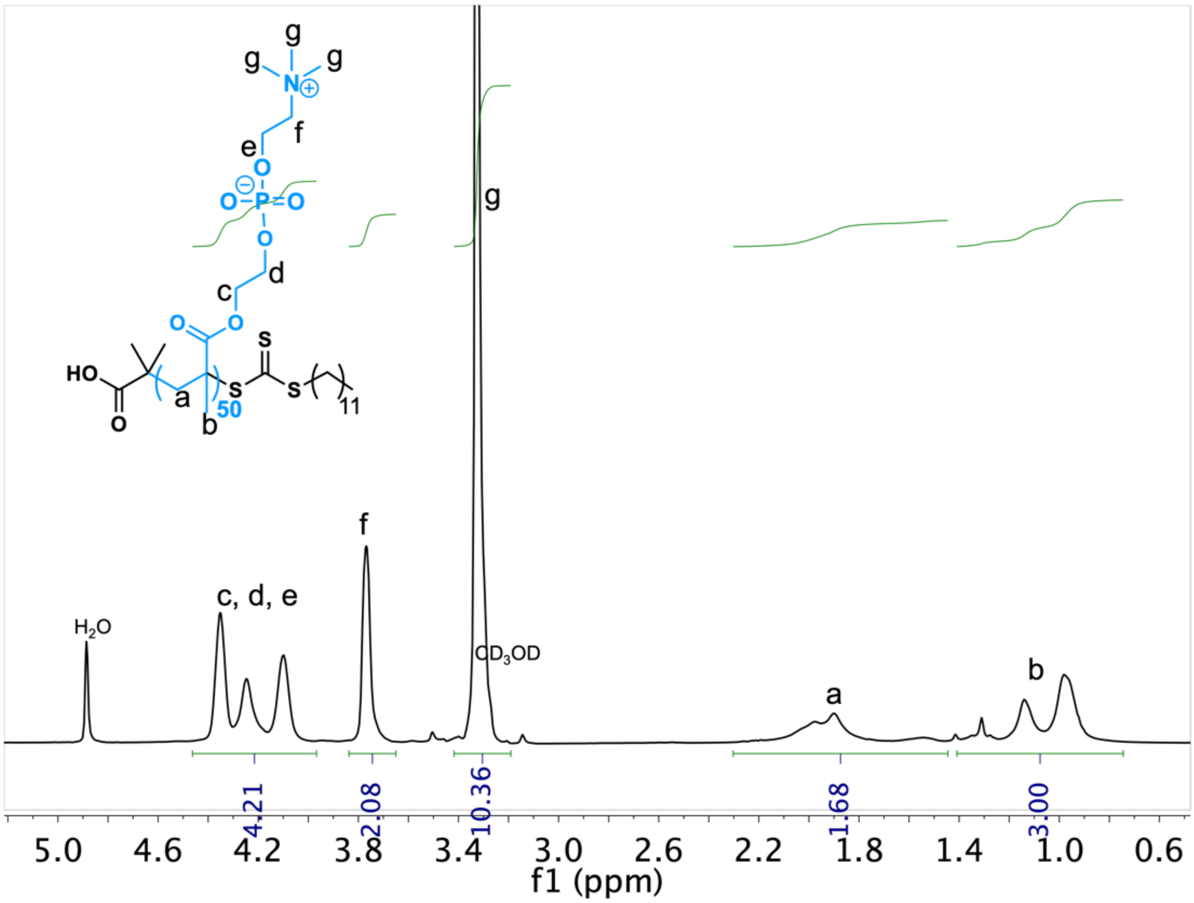

Figure S2. ^{1}H NMR spectrum for poly(2-methacryloyloxyethyl phosphorylcholine), $PMPC_{50}$ homopolymer recorded in methanol-d_4.

Photo-polymerisation of 3-[(3-acrylamidopropyl) dimethylammonio] propanoate (carboxybetaine acrylamide, CBAA) using 2-(dodecylthiocarbonothioylthio)-2-methylpropionic acid.

A vial was charged with 2-(dodecylthiocarbonothioylthio)-2-methylpropionic acid (50 mg, 0.14 mmol, 1 eq), CBAA (1.57 g, 6.86 mmol, 50 eq), and 8.1 mL of methanol. The vial was then sealed and deoxygenated using three successive cycles of freeze-pump-thaw to remove O_2(g). The sealed vial was incubated at 37°C with magnetic stirring under 460 nm light irradiation for 12h. The polymerisation was quenched by exposing the vial to air. An aliquot was withdrawn for determination of monomer conversion by ^{1}H NMR spectroscopy. The polymer was dialysed against dionised water for 72h (MWCO = 1 kDa) and subsequently

freeze-dried to yield a pale-yellow solid. $M_{n,NMR}$ was calculated by end-group analysis by comparing the integrations of the $–CH_3$ signals (t, 0.92 ppm) of methyl end-group with those of the corresponding signals of the $–CH_2$ signal (d, 3.55-3.78 ppm) of polymer backbone. ^1H NMR (400 MHz, methanol-d_4): δ (ppm) = 3.80-3.55 (br s, $NC\boldsymbol{H_2}C\boldsymbol{H_2}$ of polymer side chain), 3.55-3.18 (br m, $C\boldsymbol{H_2}CH_2C\boldsymbol{H_2}$ of polymer side chain), 3.18-2.90 (br s, $CH_2N(C\boldsymbol{H_3})_2CH_2$ of polymer side chain), 2.90-2.40 (br m, CH2$C\boldsymbol{H_2}$C(O)O) of polymer side chain), 2.40-1.20 (br m, $C\boldsymbol{H_2}C\boldsymbol{H}$ of polymer backbone and $CH_2C\boldsymbol{H_2}CH_2$ of polymer side chain), 0.92 (t, $CH_2C\boldsymbol{H_3}$ of dodecyl end group). $M_{n,NMR} = 13{,}700$ g mol^{-1} ($DP_{PCBAA, NMR} = 60$). SEC (0.1 M $NaNO_3$ in H_2O) $M_{n, SEC RI} = 6600$ g mol^{-1}, $Đ_{M, SEC RI} = 1.25$.

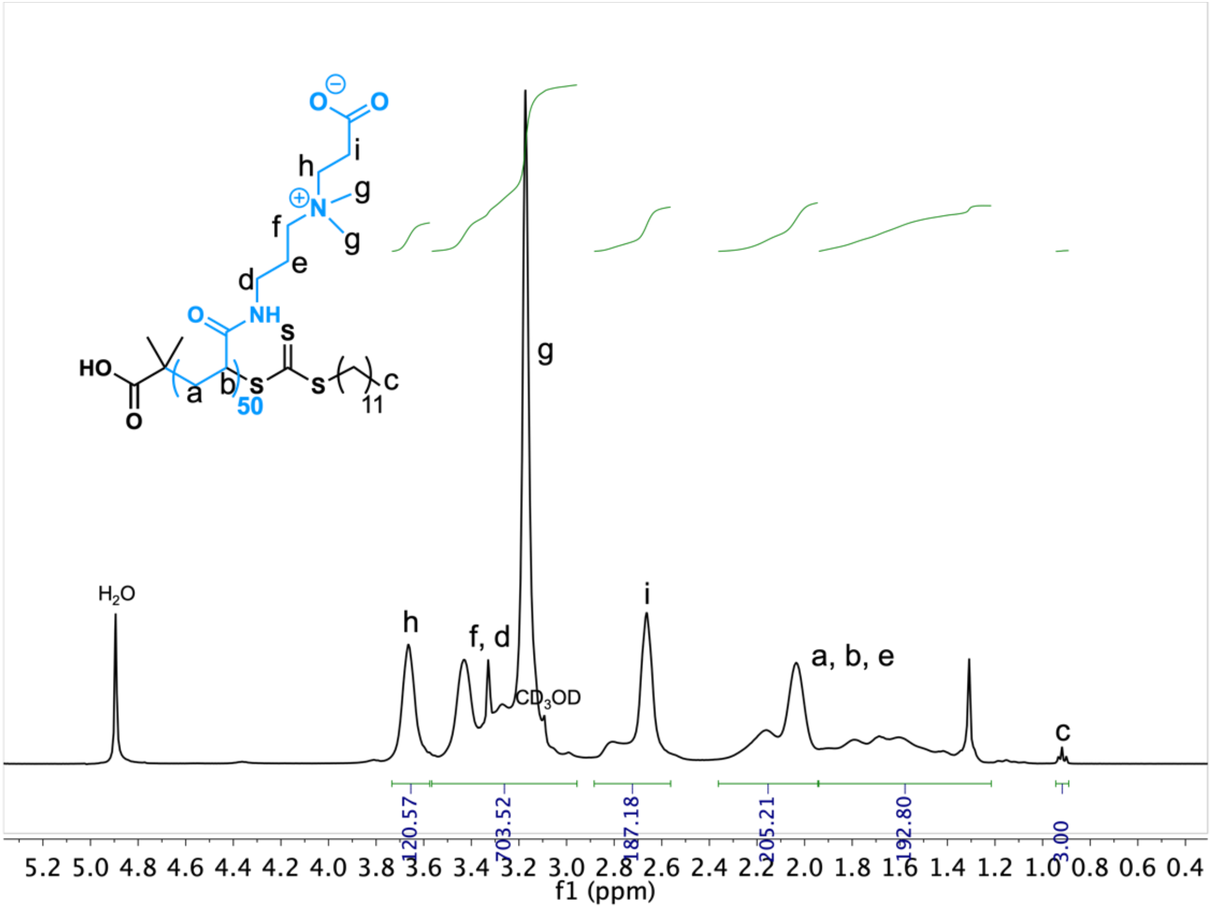

Figure S3. ^1H NMR spectrum for poly(carboxybetaine acrylamide), $PCBAA_{50}$ recorded in methanol-d_4.

Polymerisation of *N,N*-dimethylacrylamide (DMAC) using 2-(((butylthio)carbonothiolyl)thio) propanoic acid.

A vial was charged with 2-(((butylthio)carbonothiolyl)thio) propanoic acid (100 mg, 0.42 mmol, 1 eq), DMAC (1.66 g, 16.8 mmol, 40 eq), AIBN (6.9 mg, 0.04 mmol, 0.1 eq), and 7 mL

of dioxane. The vial was then sealed and deoxygenated using three successive cycles of freeze-pump-thaw to remove O_2(g). The vial was placed into an aluminium heating block which had been pre-heated to 70 °C to initiate polymerisation. After 2 h, the polymerisation was quenched by exposing the vial to air and submerging it into liquid N_2. An aliquot was withdrawn for determination of monomer conversion by ^{1}H NMR spectroscopy. The polymer was precipitated into diethyl ether from dioxane twice to yield a yellow polymer product that was further dried under vacuum. $M_{n,NMR}$ was calculated by end-group analysis by comparing the integrations of the –CH_3 signals (t, 0.97 ppm) of methyl end-group with those of the corresponding signals of the –CH signal (br m, 2.83–2.40 ppm) of polymer backbone. ^{1}H NMR (400 MHz, methanol-d_4): δ (ppm) = 3.45 (t, 2H, C(S)SC$\boldsymbol{H_2}$), 3.24-2.85 (br m, N(C$\boldsymbol{H_3}$)$_2$ of polymer side chain), 2.83-2.40 (br m, CH_2C$\boldsymbol{H}$ of polymer backbone), 1.94-1.22 (br m, CHC$\boldsymbol{H_2}$ of polymer backbone).$M_{n,NMR}$ = 4200 g mol^{-1} ($DP_{PDMAC,\ NMR}$ = 40). SEC (5 mM NH_4BF_4 in DMF) $M_{n,\ SEC\ RI}$ = 4800 g mol^{-1}, $Đ_{M,\ SEC\ RI}$ = 1.1.

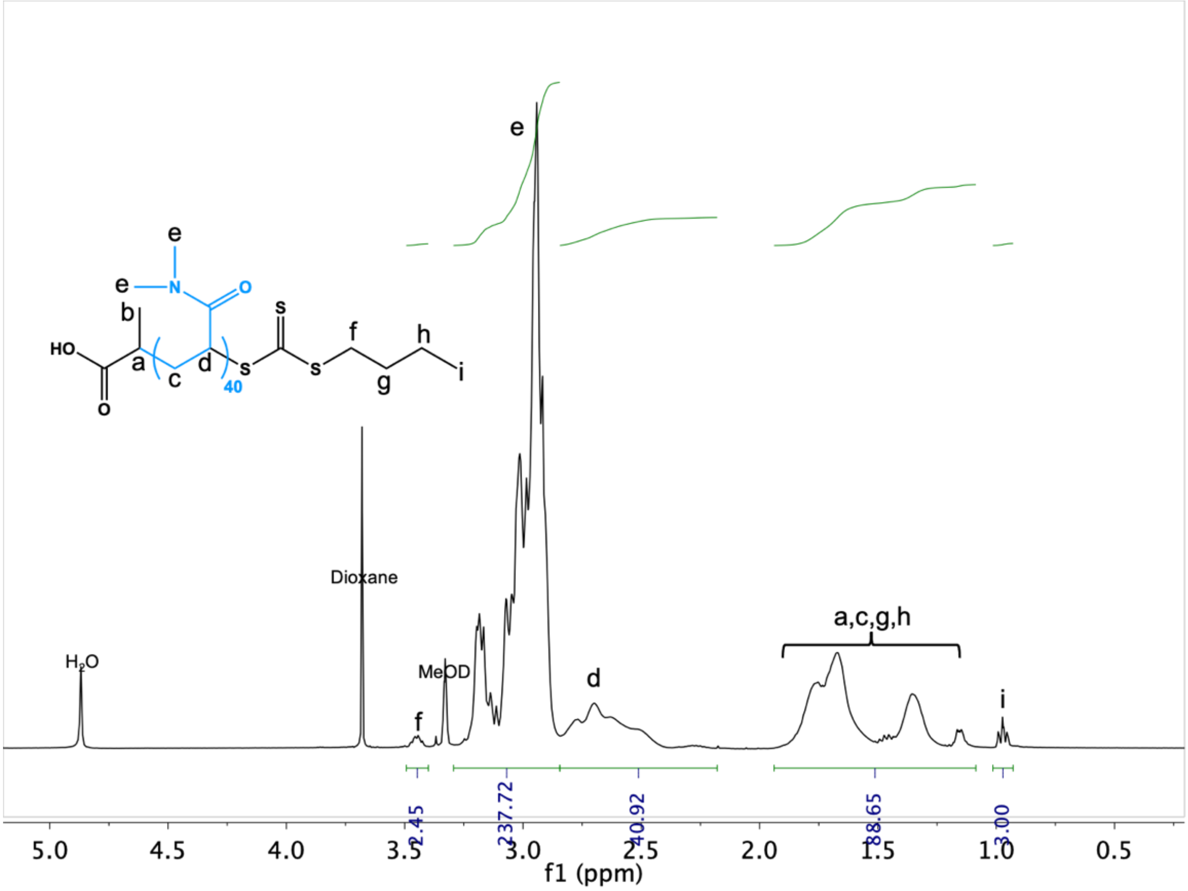

Figure S4. ^{1}H NMR spectrum for poly(dimethyl acrylamide), $PDMAC_{40}$ homopolymer recorded in methanol-d_4.

Photo-polymerisatison of *N*-(2-hydroxypropyl)methacrylamide (HPMA) using 2-(dodecylthiocarbonothioylthio)-2-methylpropanoic acid.

A vial was charged with 2-(dodecylthiocarbonothioylthio)-2-methylpropionic acid (50 mg, 0.14 mmol, 1 eq), CBAA (0.98 g, 6.86 mmol, 50 eq), and 5.2 mL of methanol. The vial was then sealed and deoxygenated using three successive cycles of freeze-pump-thaw to remove O_2(g). The sealed vial was incubated at 37 °C with magnetic stirring under 460 nm light irradiation for 12h. The polymerisation was quenched by exposing the vial to air. An aliquot was withdrawn for determination of monomer conversion by ^{1}H NMR spectroscopy. The polymer was precipitated into diethyl ether from methanol twice to yield a yellow polymer product that was further dried under vacuum. ^{1}H NMR (400 MHz. methanol-d_4): δ (ppm) 7.53 (br m, N***H*** of PHPMA side chain), 3.88 (br s, C***H*** of PHPMA side chain), 3.19- 3.02 (br m, C$\boldsymbol{H_2}$ of PHPMA sidechain), 2.05−1.79 (br m, C$\boldsymbol{H_2}$ of PHPMA backbone), 1.31−1.04 (br m, C$\boldsymbol{H_3}$ of PHPMA backbone and C$\boldsymbol{H_3}$ of PHPMA side chain), 0.92 (t, 3H, CH_2-CH_2-C$\boldsymbol{H_3}$ of dodecyl end-group). $M_{n,NMR}$ was calculated by end-group analysis by comparing the integrations of the –CH_3 signals (d, 0.92 ppm) of dodecyl end-group with those of the corresponding –CH_2 signal (br m, 2.83–2.40 ppm) of polymer backbone. $M_{n,NMR}$ = 8300 g mol^{-1} ($DP_{PHPMA, NMR}$ = 55). SEC (5 mM NH_4BF_4 in DMF) $M_{n, SEC RI}$ = 9400 g mol^{-1}, $Đ_{M, SEC RI}$ = 1.20.

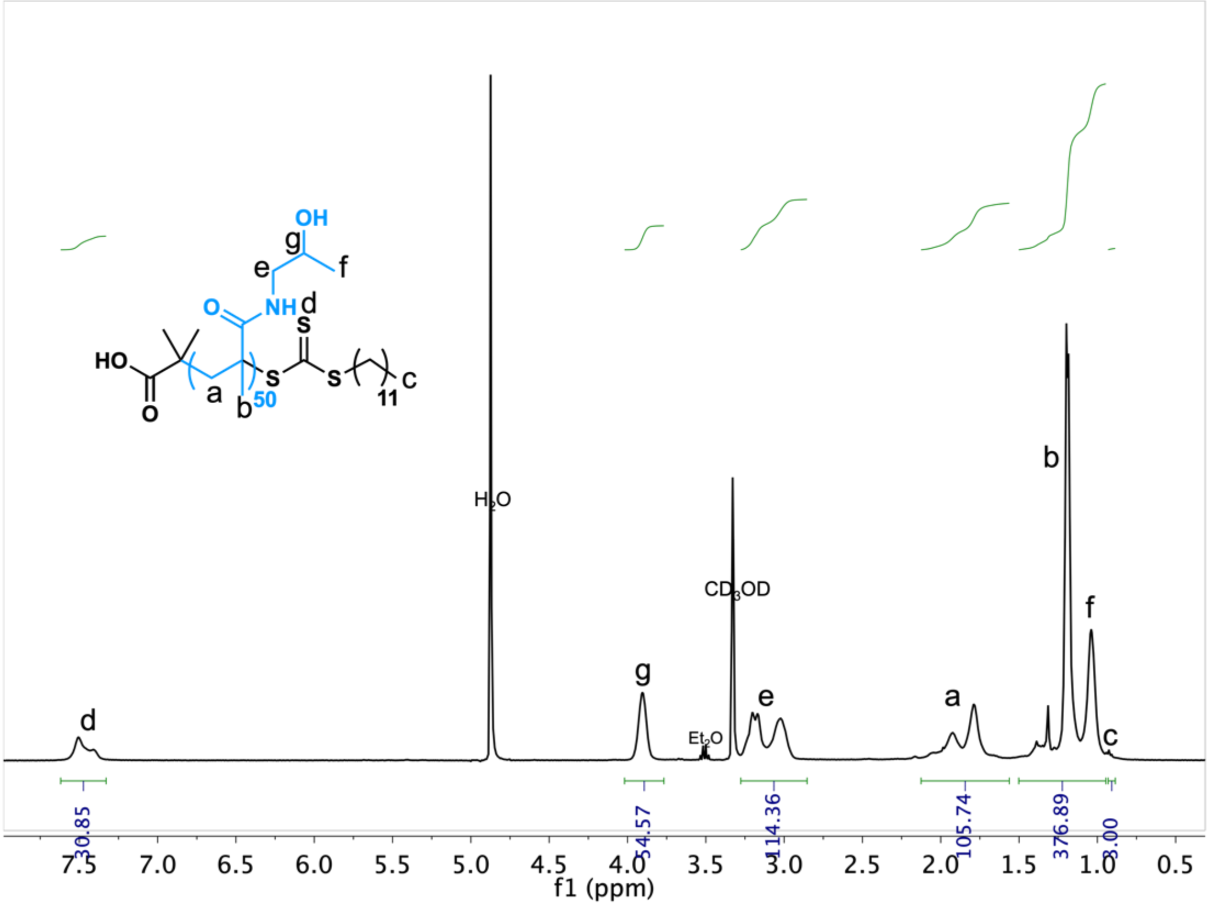

Figure S5. ^{1}H NMR spectrum for poly(*N*-(2-hydroxypropyl)methacrylamide), $PHPMA_{50}$ homopolymer recorded in methanol-d_4.

Photo-polymerisation of *N*-(2-hydroxyethyl)acrylamide (HEA) using 2-(dodecylthiocarbonothioylthio)-2-methylpropanoic acid.

A vial was charged with 2-(dodecylthiocarbonothioylthio)-2-methylpropionic acid (50 mg, 0.14 mmol, 1 eq), CBAA (0.98 g, 6.86 mmol, 50 eq), and 5.2 mL of methanol. The vial was then sealed and deoxygenated using three successive cycles of freeze-pump-thaw to remove O_2(g). The sealed vial was incubated at 37°C with magnetic stirring under 460 nm light irradiation for 12h. The polymerisation was quenched by exposing the vial to air. An aliquot was withdrawn for determination of monomer conversion by ^{1}H NMR spectroscopy. The polymer was precipitated into diethyl ether from methanol twice to yield a yellow polymer product that was further dried under vacuum. $M_{n,NMR}$ was calculated by end-group analysis by comparing the integrations of the –CH_3 signals (d, 0.85 ppm) of dodecyl end-group with those of the corresponding –CH signal (br m, 1.72-2.17 ppm) of polymer backbone. ^{1}H NMR (400 MHz. methanol-d_4): δ (ppm) 8.15-8.03 (br m, N***H*** of PHEA side chain), 3.89-3.13 (br m, NHC$\boldsymbol{H_2}$ and C$\boldsymbol{H_2}$OH of PHEA side chain), 2.35−2.05 (br m, C***H*** of PHEA backbone), 1.85-

1.31 (br m, C$\boldsymbol{H_2}$ of PHEA backbone), 0.92 (t, 3H, $CH_2C\boldsymbol{H_3}$ of dodecyl end-group). $M_{n,NMR}$ = 6800 g mol^{-1} ($DP_{PHEA, NMR}$ = 56). SEC (5 mM NH_4BF_4 in DMF) $M_{n, SEC RI}$ = 9300 g mol^{-1}, $Đ_{M, SEC RI}$ = 1.14. Same procedure was followed for the synthesis of pentafluorophenyl-end PHEA telechelic homopolymers using 2-(dodecylthiocarbonothioylthio)-2-methylpropanoic acid pentafluorophenyl ester (PFP-DMP) using [HEA]:[CTA] ratios of 25, 50 and 75.

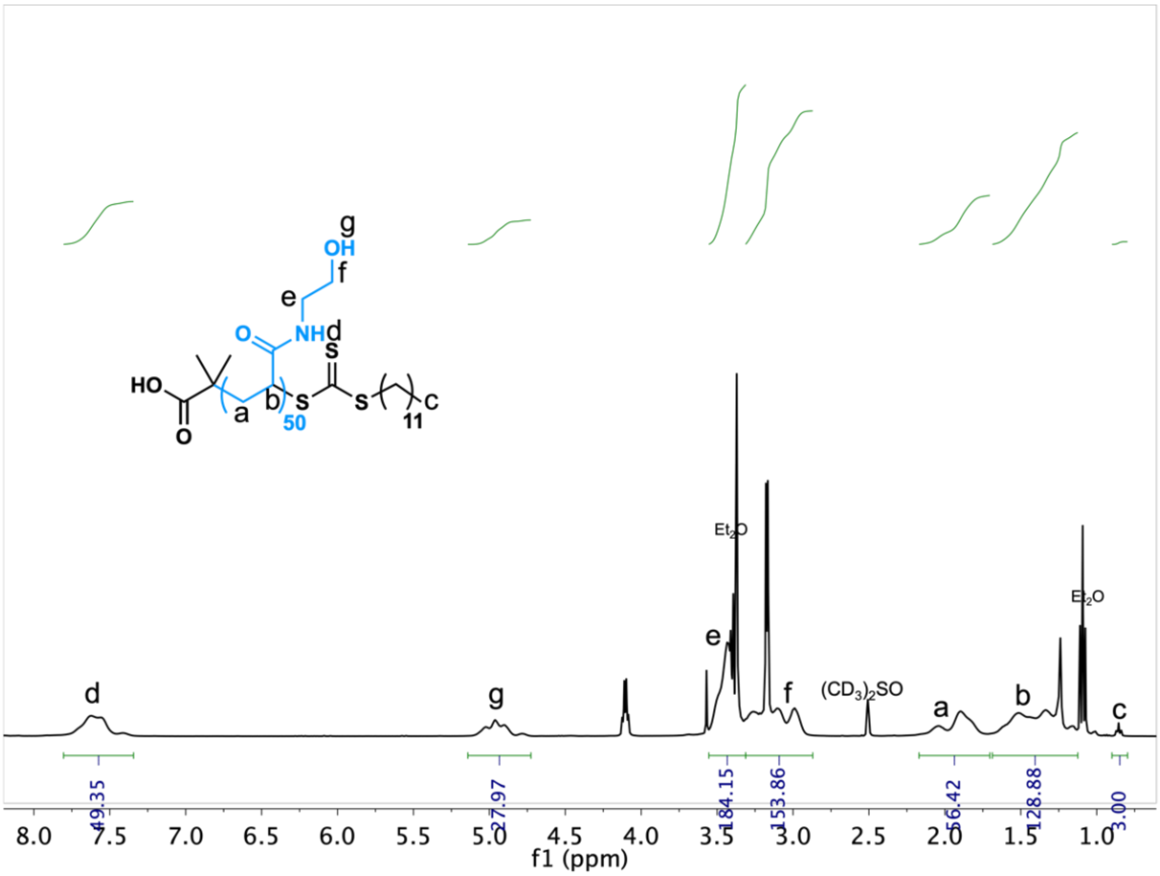

Figure S6. ^{1}H NMR spectrum for poly(*N*-(2-hydroxyethyl)acrylamide), $PHEA_{50}$ homopolymer recorded in DMSO-d_6.

End-group modification of PFP-poly(*N*-hydroxyethyl acrylamide) (PFP-PHEA) homopolymers using galactosamine.

In a typical reaction, PFP-$PHEA_{25}$ (100 mg, 0.011 mmol), galactosamine (11.4 mg, 0.053 mmol) were dissolved in 5 mL DMF with 0.05 M triethylamine (TEA) (50 µL). The reaction was stirred at 50 ˚C for 16 hrs. The polymer was precipitated into diethyl ether from methanol three times and dried over under vacuum. ^{19}F-NMR and IR analysis were performed and confirmed the loss of the pentafluoro end-group. Same procedure was followed for the synthesis of Gal-$PHEA_{50}$ and Gal-$PHEA_{75}$.

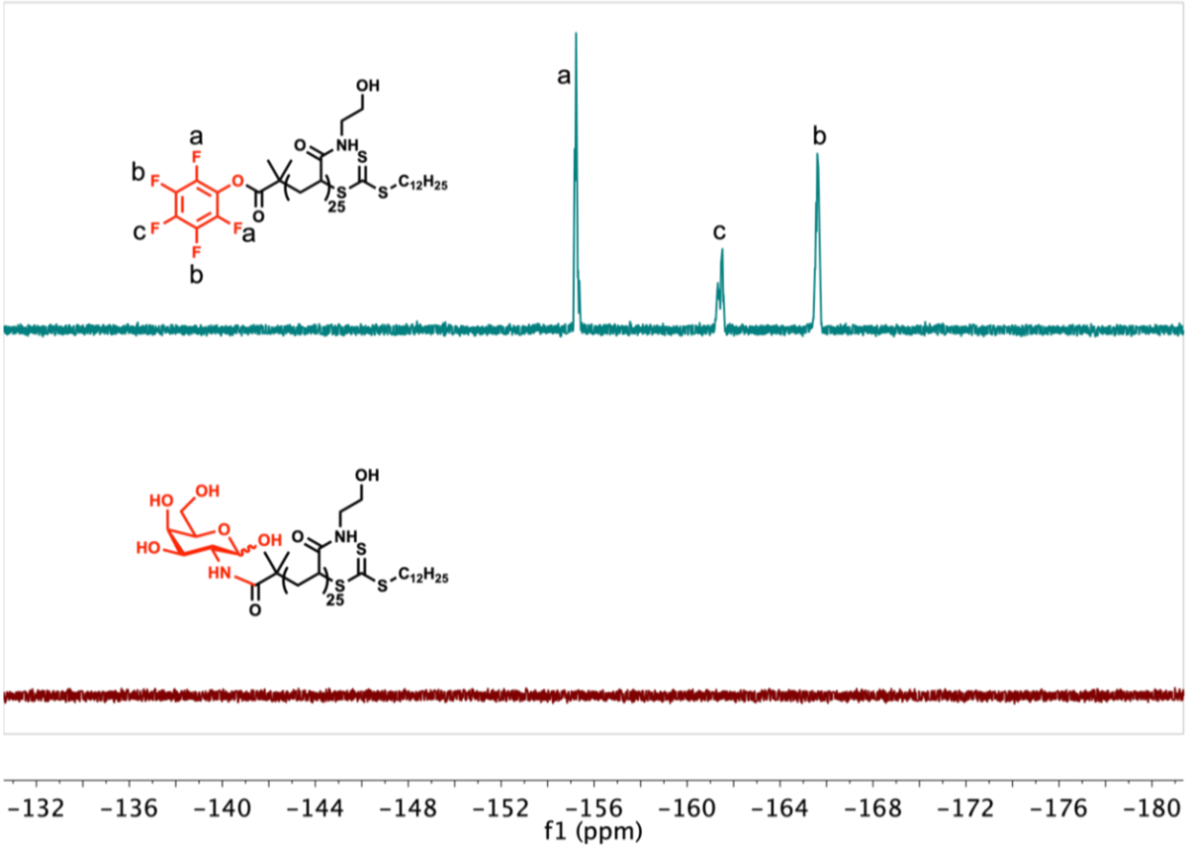

Figure S7. ^{19}F NMR spectra for the purified PFP/Gal-PHEA$_{25}$ homopolymer before and after post-functionalisation with D-(+)-galactosamine. All spectra were recorded in methanol-d_4.

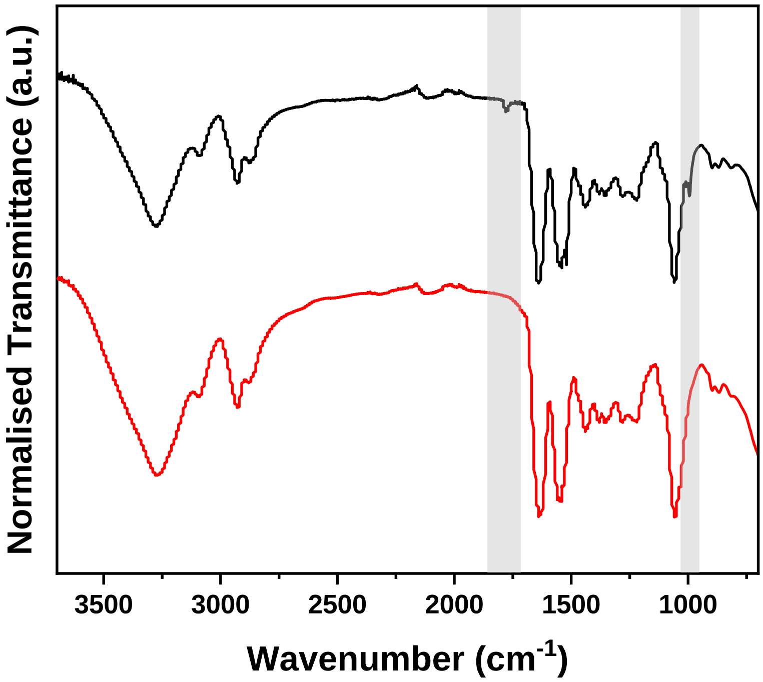

Figure S8. FT-IR spectra for PHEA$_{25}$ homopolymer before (black) and after (red) end-group modification with D-(+)-galactosamine. The disappearance of the characteristic vibration peaks of PFP group at 950 and 1750 cm^{-1} is shown.

Gold nanoparticle functionalization. Approximately 1 mg of the desired polymer was added to a micro-centrifuge tube and dissolved in 100 μL of high-purity water. 900 μL of the citrate-stabilised gold nanoparticle solution was added to this tube (40 nm NP solution) that was then agitated for 30 mins in the absence of light. To remove excess polymer, the particles were centrifuged and following careful removal of the supernatant, the particles were then redispersed in 1 mL of MilliQ water, and the centrifugation-resuspension process repeated for a total of 3 cycles. After the final cycle the particles were dispersed in 1 mL of MilliQ water for future use. TEM, DLS and zeta-potential analyses were performed on the samples after dilution to an appropriate analysis concentration.

Formation of biomolecular corona

Plasma was reconstituted in ultrapure water and filtered through a 0.22 um syringe filter. The filtered plasma was aliquoted into 2 ml cryo-tubes and stored −80°C until use. For formation of biomolecular corona, an aliquot of the plasma from −80°C was first defrosted at room temperature and then diluted to a protein concentration of 10% or 80% (v/v) with pure water. This concentration was close to the in *vitro and in vivo* plasma protein concentration in cell culture and blood, respectively. For the assay, 250 μL of the gold nanoparticles (~1.0 final OD) were incubated with an equal volume (1:1) of the diluted plasma for 1 hour at 37°C, and 300 rpm. The unbound plasma proteins were removed by centrifugation at 12000 g, 20°C for 20 min. The supernatant, containing unbound plasma proteins, were discarded and pellet, containing gold nanoparticles-plasma complex, was resuspended in 400 μL PBS and centrifuged again. The washing step was repeated three times to ensure removal of soft corona proteins i.e., loosely bound proteins. After each centrifugation step, 10 μL of the supernatant (containing the released soft corona proteins) was collected for SDS-PAGE analysis. After the final wash, the hard-corona coated gold nanoparticles were either resuspended in *NuPAGE™ LDS Sample Buffer (2x)* (containing 50 mM DTT) for SDS-PAGE analysis or resuspended in desired volume of assay buffer (10 mM HEPES, 150 mM NaCl, 10 mM, 10 mM $CaCl_2$) for lectin binding assays.

BSA blocking. The globulins free BSA for blocking functionalised gold nanoparticles, prior to incubation in bovine plasma, was used at two different concentrations i.e., 2.5% and 5% (v/v). Briefly, 250μL of gold nanoparticles solution was incubated with an equal volume (1:1) of either 2.5% or 5% BSA for 1 hour at 37°C, and 300 rpm. The Gal-PHEA@AuNPs-BSA

complex was centrifuged for removing the unbound BSA proteins at 12000 g, 20°C for 20 min. After centrifugation, the supernatant was discarded, and pellet was resuspended in 400 μL of PBS and centrifuged again. The washing step was repeated at least three times to completely remove the loosely bound BSA molecules. After final wash, the Gal-PHEA@AuNPs-BSA complex was resuspended in 250 μL of PBS and incubated with an equal volume (1:1) of the 80% bovine plasma for 1 hour at 37°C, and 300 rpm. After incubation, the samples were processed using a similar protocol as described earlier in the section "Formation of biomolecular corona".

Characterisation of biomolecular corona

Sodium Dodecyl Sulphate–Polyacrylamide Gel Electrophoresis (SDS-PAGE). The SDS-PAGE technique was used to detect and separate the soft and hard corona proteins present on the surface of gold nanoparticles. Briefly, the protein corona coated AuNPs were resuspended in 20 μL of PBS and mixed with 20 μL of *NuPAGE™ LDS Sample Buffer (2x)* containing 50 mM DTT. The solution was heated at 70 °C for 5 min, to speed up the denaturation process, and loaded into NuPAGE™ 12% Bis-Tris Precast Gels. The Gel was run at 180V for 35 min in NuPAGE™ (1*x*) MOPS SDS Running Buffer using NuPAGE Mini Gel Tank.

Coomassie staining. After the run, the gel was rinsed with distilled water and placed in ~15 mL of InstantBlue® Coomassie Protein Stain and left overnight on a shaker for visualizing protein bands.

Silver staining. For silver staining, the gel after electrophoresis was rinsed with distilled water and then placed in a 60 mL of fixer solution (50% acetone, 1.5 ml of 50% trichloroacetic acid, 25 µL of 37% formaldehyde) for 15 mins on a shaker. Followed by washing with distilled water (1 x 5 min, and 3 x 1 min). The gel was then washed with 60 mL of 50% acetone solution for 5 minutes on a shaker, and then quickly put in 60 mL of pre-stain solution (10 mg of sodium thiosulfate) for 1 minute. Further, the gel was washed with distilled water (3 x 1 min) and put in a staining solution (160 mg silver nitrate, 600 µl of 37% formaldehyde) for 8 minutes. The gel was then washed with distilled water (2 x 1 min) and the bands were developed by putting the gel in 60 mL of developer solution (1.2 g Na_2CO_3, 25 µl Formaldehyde, 2.5 mg sodium thiosulfate) for 10-20 seconds. Finally, the gel was placed in stopping solution (1% Acetic Acid) for 2 minutes, and then rinsed with distilled water.

Western blotting. In order to detect specific glycans/sugars attached to glycoproteins in the hard corona samples, western blotting was performed. The gel from SDS-PAGE was first rinsed with distilled water and the proteins along with the prestained ladder were transferred into the polyvinylidene fluoride (PVDF) membrane, using Trans-Blot® Turbo™ Mini polyvinylidene fluoride (PVDF) Transfer Pack (Bio-Rad, UK) as per the manufacturer's instructions. The Trans-Blot® Turbo™ Transfer System (Bio-Rad) was used to transfer the proteins using the protocol i.e., 1.3 A, 21 V, 7 min. The SiaFind™ Pan-Specific Lectenz® Kit was then used, to detect the terminal sialic acids present on glycoproteins transferred to the membrane, as per the manufacturer's instructions.

Lectin binding assays

Bio-layer Interferometry (BLI). The Octet® RED96 Bio-Layer Interferometry system (Forte Bio, USA) with Octet® Streptavidin (SA) or NTA Biosensors was used for lectin binding studies, at 30°C with shaking at 1000 rpm. For SA biosensors, the lectins (SBA, WGA, MAL II) were first biotinylated using the EZ-Link™ Sulfo-NHS-LC-Biotin kit as per the manufacturer's instruction. The NTA Biosensors were used for polyhistidine tagged human Siglec-2/CD22 lectin. The assay buffer was 0.22 μm filtered and consisted of 10 mM HEPES, 150 mM NaCl, and 10 mM $CaCl_2$ at pH 7.4. The biosensors were pre-hydrated in 200 μL of BLI assay buffer for at least 10 min in the biosensor's plate to remove the protective sucrose coating. Flat bottom black 96-Well microplates were used and loaded with 200 μL of liquid per well. The assay plate was prepared as follow, column 1 (assay buffer), column 2 (100-200 μg/mL of the lectin in assay buffer), column 3 (assay buffer), column 4 (nanoparticles in assay buffer at final OD ~1.0), and column 5 (assay buffer). Furthermore, the BLI assay was carried out as follow, Baseline 1 (120 s) in column 1 (Equilibration), loading (300 s) in column 2 (immobilization of the lectin on the biosensor), baseline 2 (120 s) in column 3 (wash off loosely bound lectins), binding/association (2000 s) in column 4 (immobilized lectin binding to gold nanoparticles in solution), and finally dissociation (600 s) in column 5 (wash off loosely bound complexes).

Lectin-induced aggregation by absorbance. A stock solution of the lectin (SBA and WGA) was prepared at 2 mg/mL (~16 uM) concentration in assay buffer. 25 μL serial dilution was made up in the same buffer in a clear, flat bottom, half area 96-well microtiter plate. Gold nanoparticles (25 μL, ~1,0 final OD) in assay buffer were added to each well and the plate was

gently agitated at room temperature for 30 min. The absorbance spectra were recorded from 400 to 700 nm with 1 nm interval using a Biotek Synergy HT micro-plate reader.

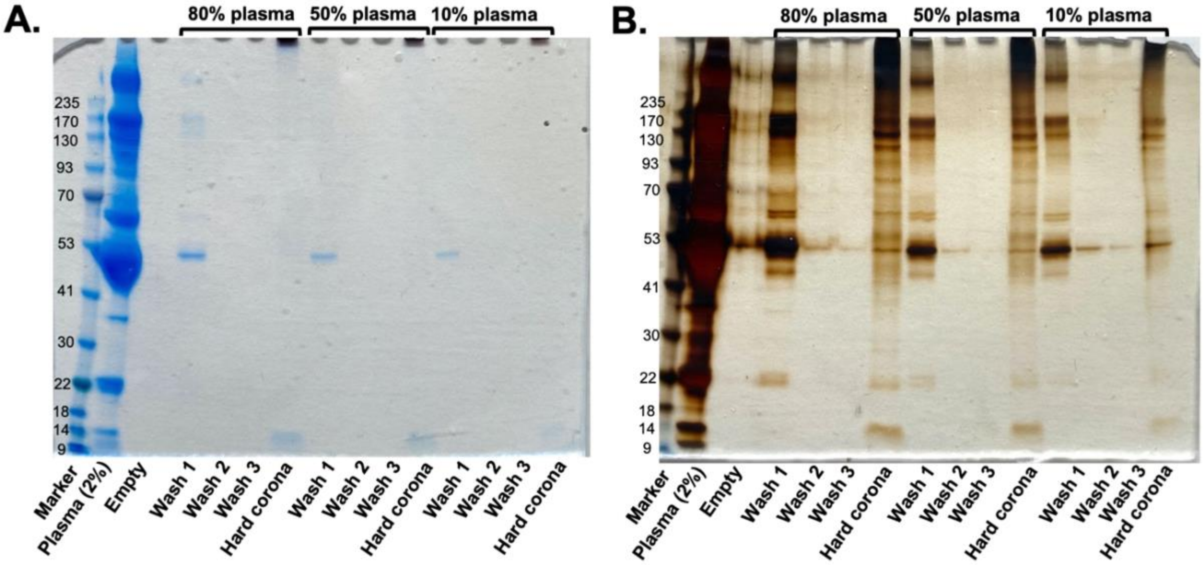

Figure S9. SDS-PAGE of protein corona formation on naked gold nanoparticles (AuNPs). A) Coomassie-stained gel showing little, or no soft (Wash 1-3) and hard corona proteins released from AuNPs after incubation with bovine plasma; B) Silver-stained gel of the same AuNPs samples showing large amount of soft and hard corona proteins. Plasma (2%) is the diluted plasma loaded into the gel.

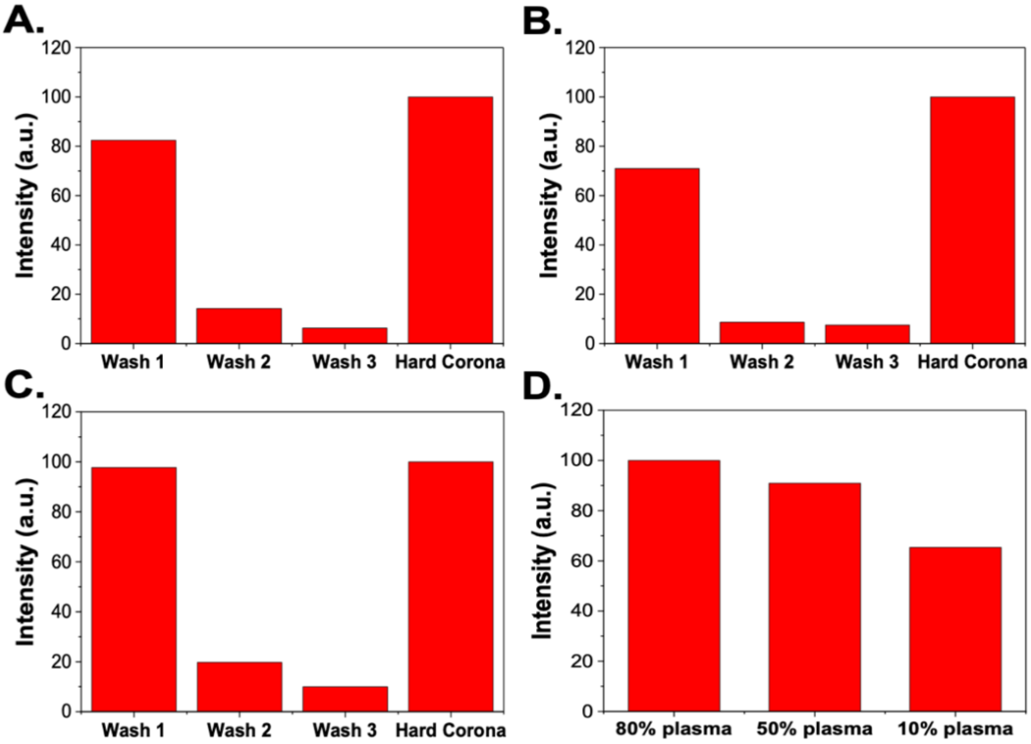

Figure S10. Densitometry analysis of the gel of protein corona (soft and hard) from naked AuNPs at different bovine plasma concentrations. A) 80% plasma; B) 50%; C) 10%; D) Hard corona at different plasma concentrations.

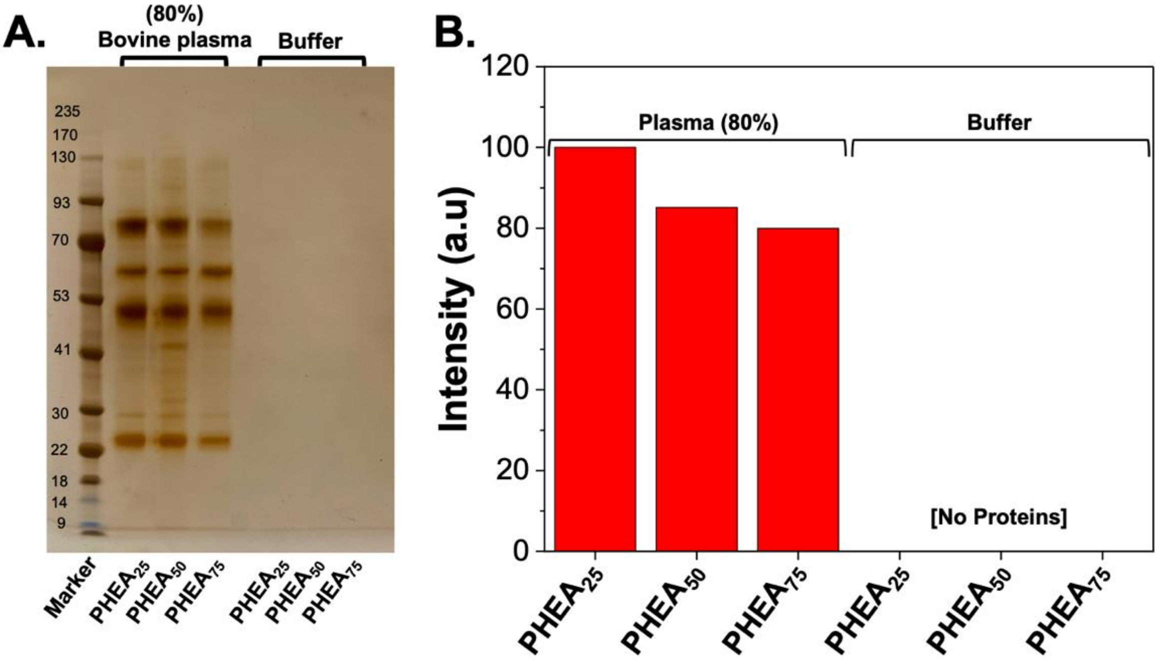

Figure S11. SDS-PAGE of protein corona formation on Gal-PHEA$_n$@AuNPs (n = 25, 50,75). A) Silver-stained gel showing protein corona profile of nanoparticles after incubation with *Bovine Plasma* (lane 2-4), and *buffer* (lane 5-7); B) Densitometry analysis of gel. Polymer codes refer to polymer coatings (Figure 2) on 40 nm gold particles.

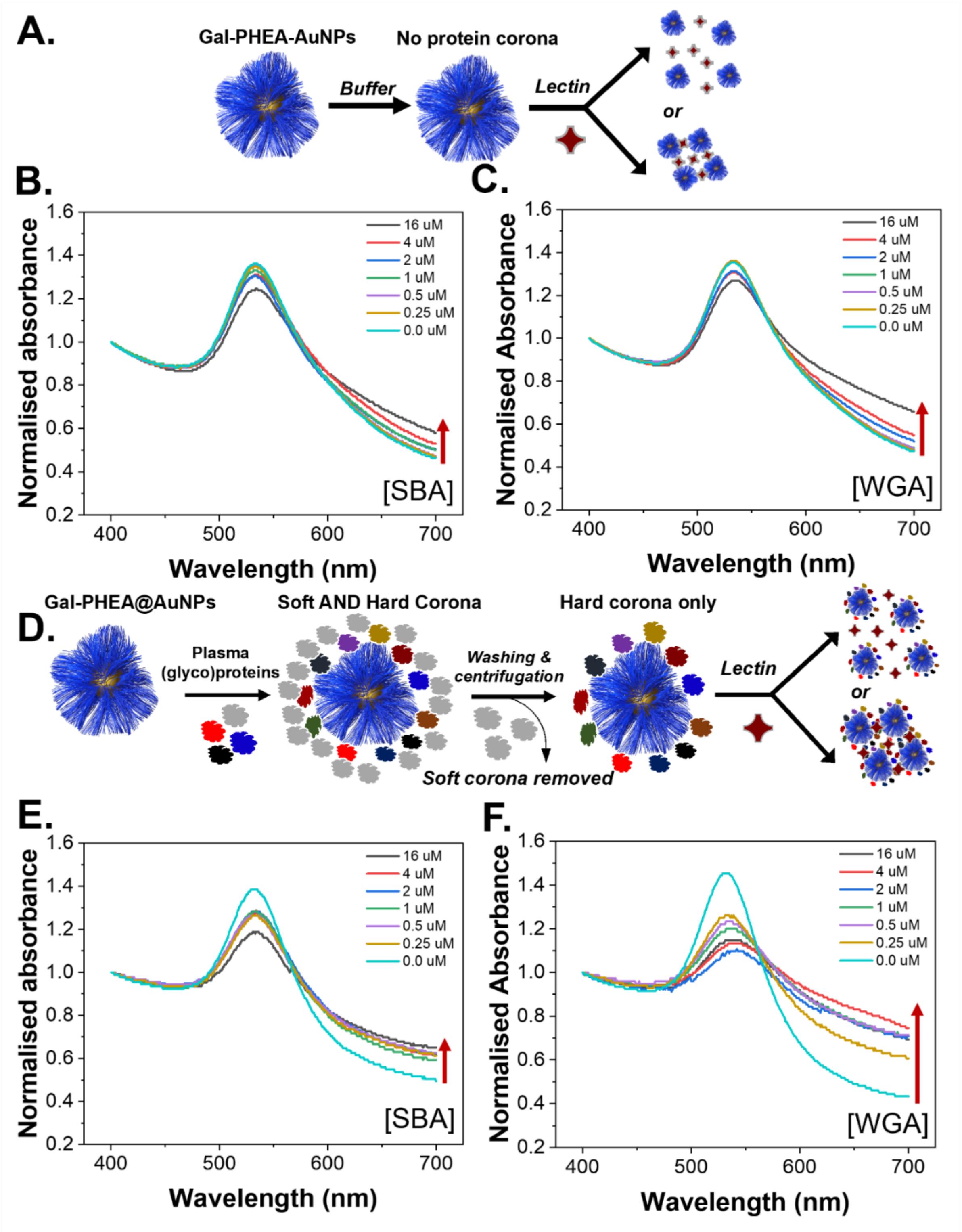

Figure S12. Effect of hard corona on lectin binding capacity to Gal-PHEA$_{50}$@AuNPs. A) Schematic of aggregation assay in buffer only; B) UV-Vis spectra verses SBA in buffer; C) UV-vis spectra verses WGA in buffer; D) Schematic of hard corona formation and aggregation assay; E) UV-Vis spectra verses SBA with hard corona; E) UV-vis spectra verses WGA with hard corona.

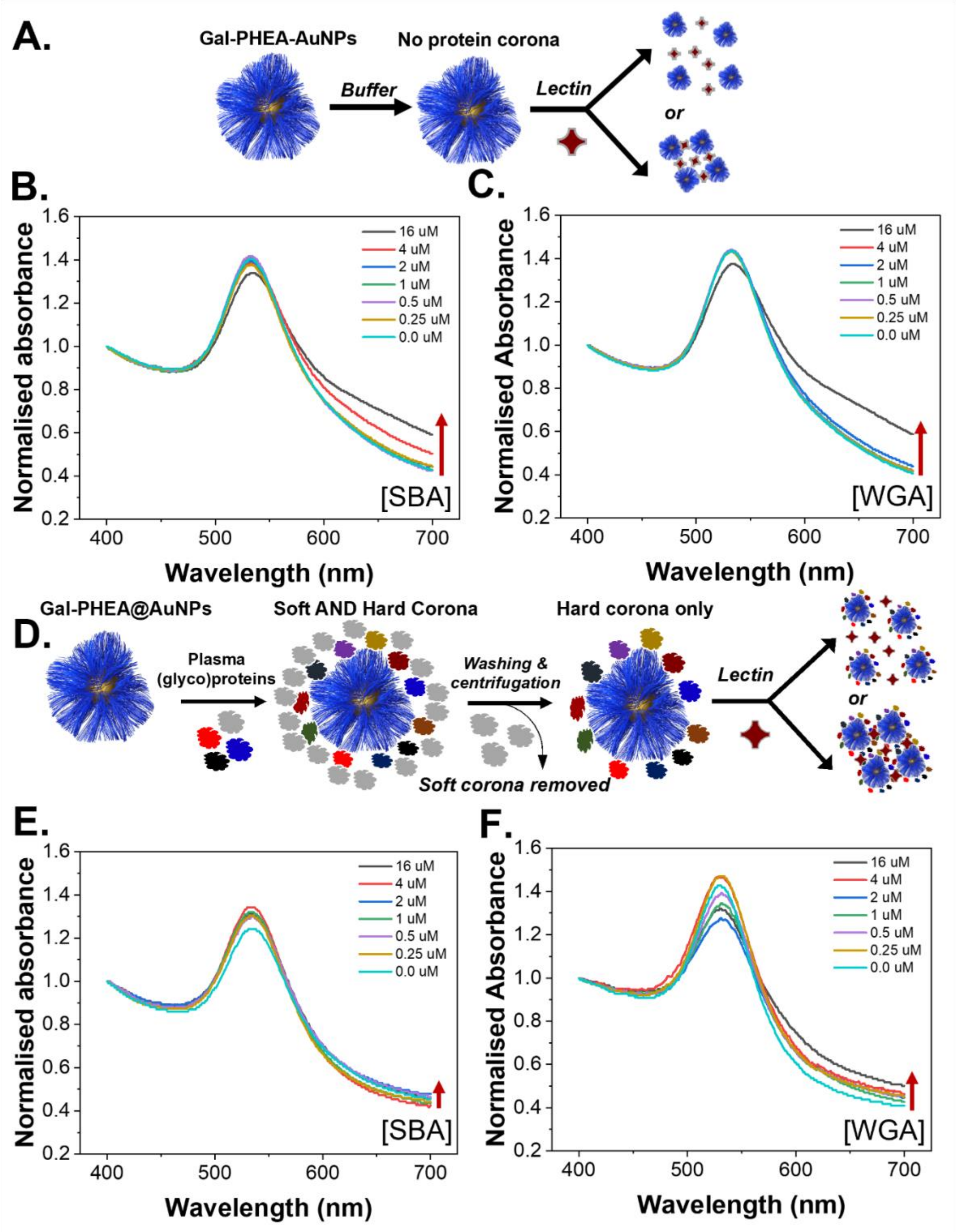

Figure S13. Effect of hard corona on lectin binding capacity to Gal-PHEA$_{75}$@AuNPs. A) Schematic of aggregation assay in buffer only; B) UV-Vis spectra verses SBA in buffer; C) UV-vis spectra verses WGA in buffer; D) Schematic of hard corona formation and aggregation assay; E) UV-Vis spectra verses SBA with hard corona; E) UV-vis spectra verses WGA with hard corona.

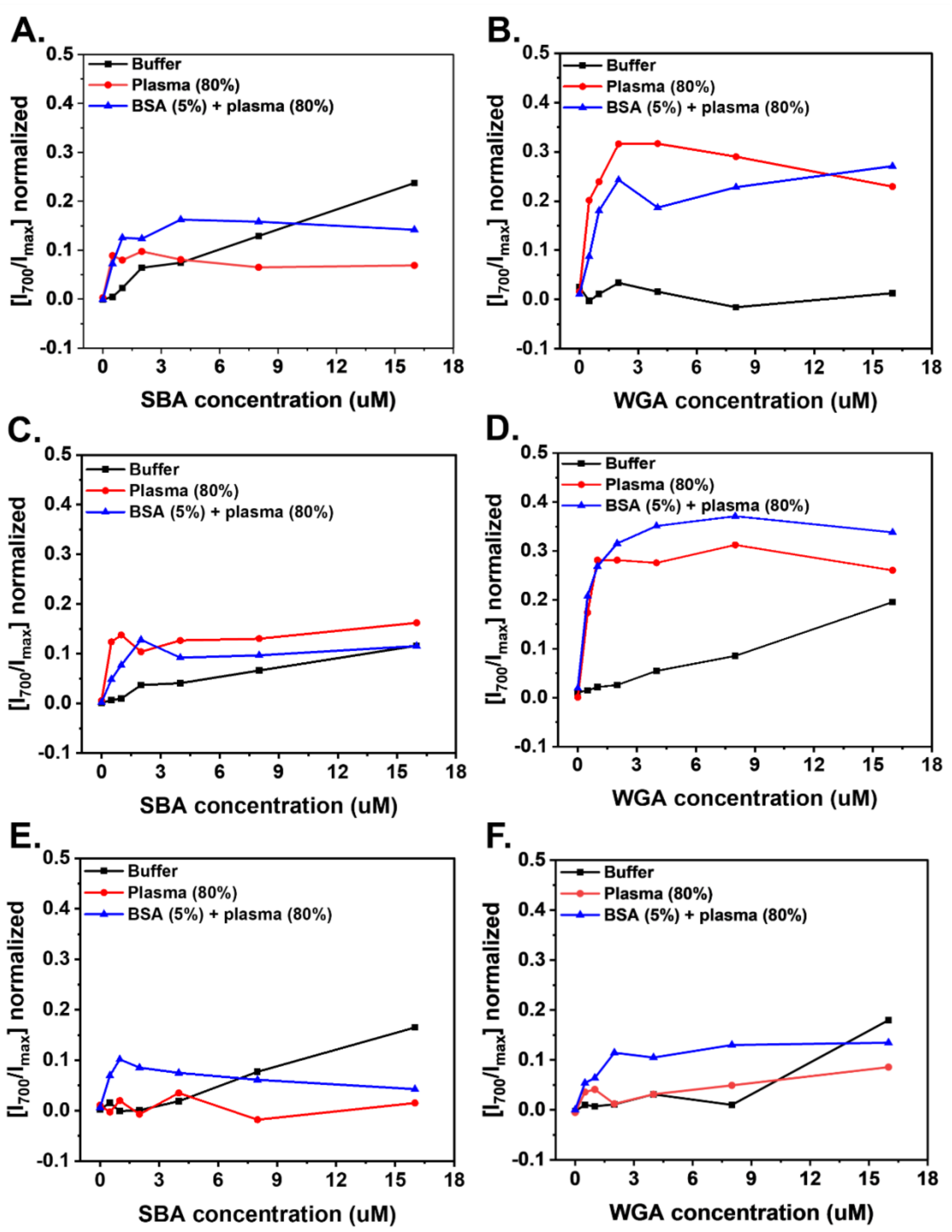

Figure S14. Binding isotherms of Gal-PHEA$_n$@AuNPs (n = 25, 50, 75) with soybean agglutinin (SBA) and wheat germ agglutinin (WGA) in three different conditions (i.e., buffer alone, plasma alone, and BSA then plasma) determined by UV-visible spectroscopy. (A) Gal-PHEA$_{25}$@AuNPs verses SBA; (B) PHEA$_{25}$@AuNPs verses WGA; (C) PHEA$_{50}$@AuNPs verses SBA; (D) PHEA$_{50}$@AuNPs verses WGA; (E) PHEA$_{75}$@AuNPs verses SBA; (F) PHEA$_{75}$@AuNPs verses WGA. All samples were incubated for 30 min at 37°C. Y-Axis is ratio of absorbance at 700 nm, relative to the SPR maximum wavelength of the Gal-PHEA@AuNPs.

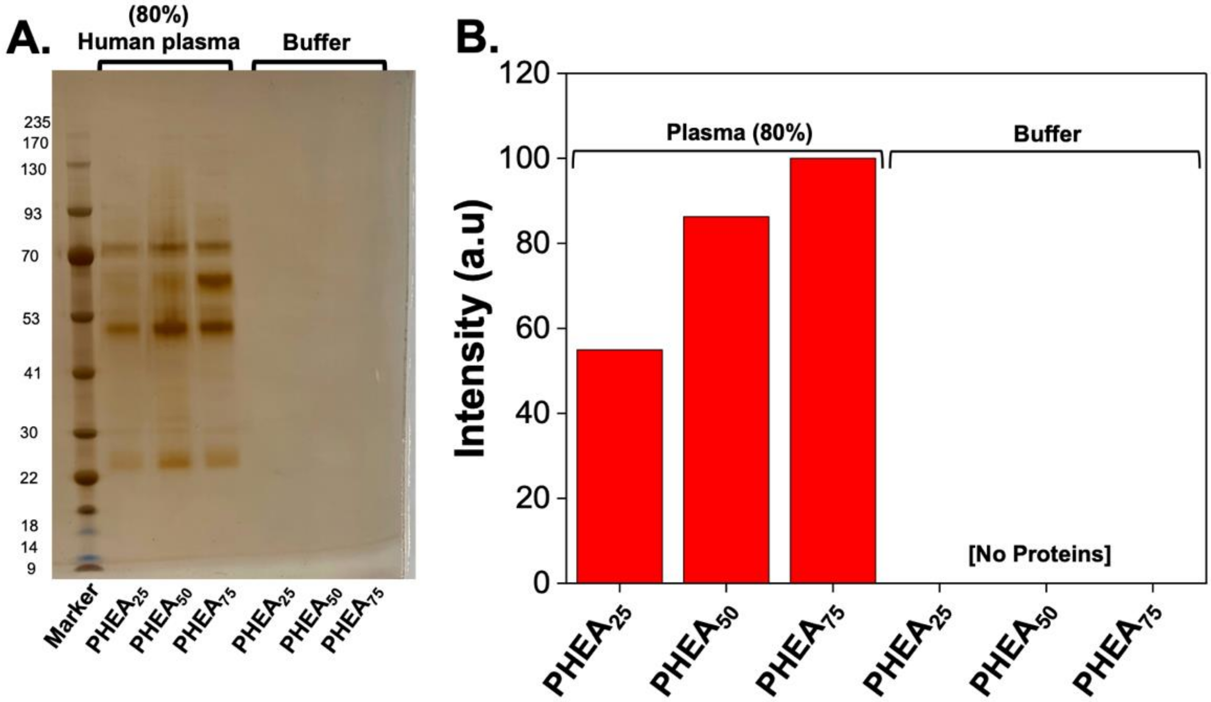

Figure S15. SDS-PAGE of protein corona formation on Gal-PHEA$_n$@AuNPs (n = 25, 50,75). A) Silver-stained gel showing protein corona profile of nanoparticles after incubation with *Human Plasma* (lane 2-4), and *buffer* (lane 5-7); B) Densitometry analysis of gel. Polymer codes refer to polymer coatings (Figure 2) on 40 nm gold particles.

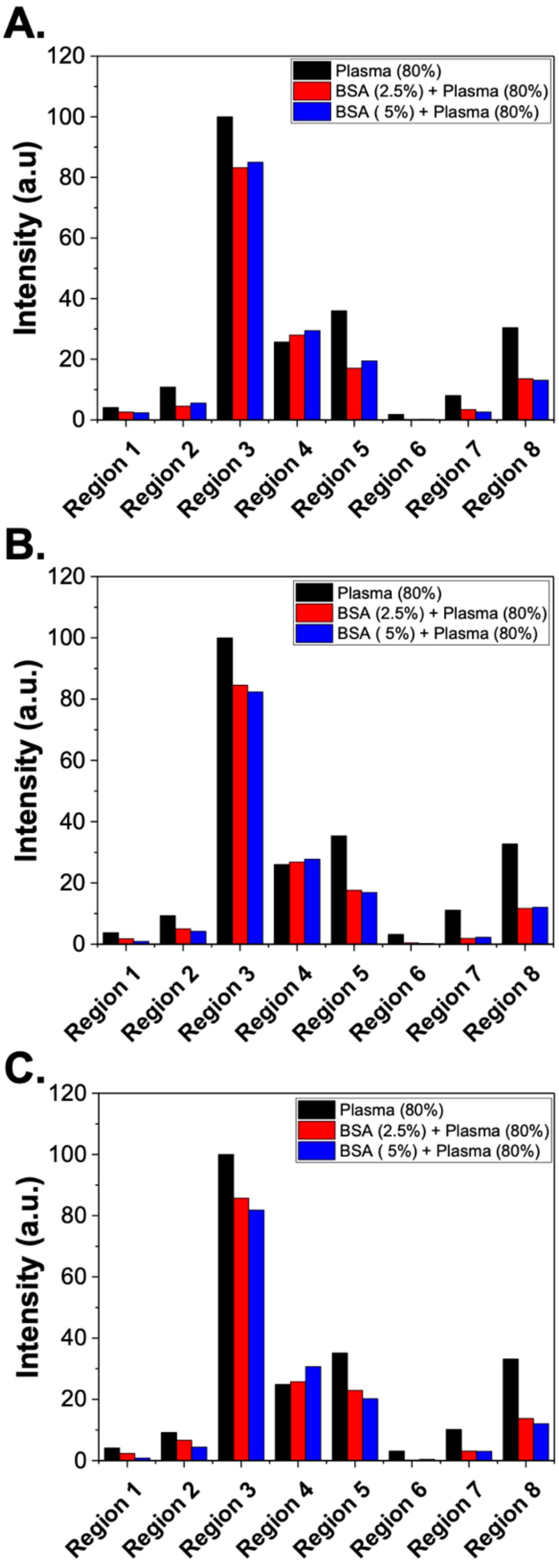

Figure S16. Densitometry analysis of hard corona proteins (region 1-8) on the silver-stained gel from Gal-PHEA@AuNPs at three different conditions. A) Gal-PHEA$_{25}$@AuNPs; B) Gal-PHEA$_{50}$@AuNPs; C) Gal-PHEA$_{75}$@AuNPs.

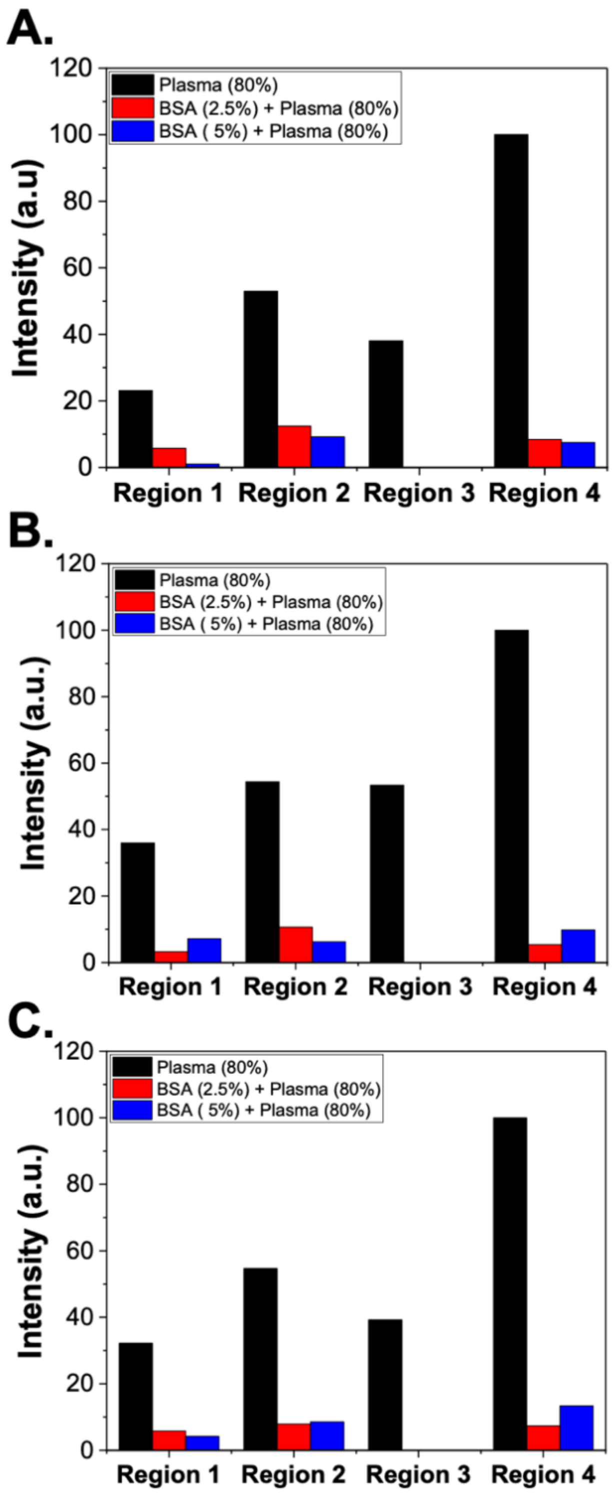

Figure S17. Densitometry analysis of sialic acid contents on glycoproteins (region 1-4) from the western blot of Gal-PHEA@AuNPs at three different conditions. A) Gal-PHEA$_{25}$@AuNPs; B) Gal-PHEA$_{50}$@AuNPs; C) Gal-PHEA$_{75}$@AuNPs.

References

1 C. A. Bell, G. G. Hedir, R. K. O'Reilly and A. P. Dove, *Polym. Chem.*, 2015, **6**, 7447–7454.

2 C. J. Ferguson, R. J. Hughes, D. Nguyen, B. T. T. Pham, R. G. Gilbert, A. K. Serelis, C. H. Such and B. S. Hawkett, *Macromolecules*, 2005, **38**, 2191–2204.

3 P. G. Georgiou, H. L. Marton, A. N. Baker, T. R. Congdon, T. F. Whale and M. I. Gibson, *J. Am. Chem. Soc.*, 2021, **143**, 7449–7461.

4 Ž. Krpetić, A. M. Davidson, M. Volk, R. Lévy, M. Brust and D. L. Cooper, *ACS Nano*, 2013, **7**, 8881–8890.

5 J. A. Jamison, K. M. Krueger, C. T. Yavuz, J. T. Mayo, D. LeCrone, J. J. Redden and V. L. Colvin, *ACS Nano*, 2008, **2**, 311–319.

6 M. P. Monopoli, D. Walczyk, A. Campbell, G. Elia, I. Lynch, F. Baldelli Bombelli and K. A. Dawson, *J. Am. Chem. Soc.*, 2011, **133**, 2525–2534.

7 A. S. Pitek, D. O'Connell, E. Mahon, M. P. Monopoli, F. Francesca Baldelli and K. A. Dawson, *PLoS One*, 2012, **7**, e40685.

8 A. Perez-Potti, H. Lopez, B. Pelaz, A. Abdelmonem, M. G. Soliman, I. Schoen, P. M. Kelly, K. A. Dawson, W. J. Parak, Z. Krpetic and M. P. Monopoli, *Sci. Rep.*, 2021, **11**, 6443.
